# Multiplex coherent anti-Stokes Raman scattering microspectroscopy detection of lipid droplets in cancer cells expressing TrkB

**DOI:** 10.1038/s41598-020-74021-z

**Published:** 2020-10-07

**Authors:** Tiffany Guerenne-Del Ben, Vincent Couderc, Ludovic Duponchel, Vincent Sol, Philippe Leproux, Jean-Michel Petit

**Affiliations:** 1 PEIRENE, EA 7500, University of Limoges, 123 Avenue Albert Thomas, 87060 Limoges, France; 2 XLIM, UMR 7252, University of Limoges, 123 Avenue Albert Thomas, 87060 Limoges, France; 3 Univ. Lille, CNRS, UMR 8516-LASIR-Laboratoire de Spectrochimie Infrarouge et Raman, 59000 Lille, France; 4 LEUKOS, 37 rue Henri Giffard, 87280 Limoges, France

**Keywords:** Biophysics, Cancer, Cell biology

## Abstract

For many years, scientists have been looking for specific biomarkers associated with cancer cells for diagnosis purposes. These biomarkers mainly consist of proteins located at the cell surface (e.g. the TrkB receptor) whose activation is associated with specific metabolic modifications. Identification of these metabolic changes usually requires cell fixation and specific dye staining. MCARS microspectroscopy is a label-free, non-toxic, and minimally invasive method allowing to perform analyses of live cells and tissues. We used this method to follow the formation of lipid droplets in three colorectal cancer cell lines expressing TrkB. MCARS images of cells generated from signal integration of CH_2_ stretching modes allow to discriminate between lipid accumulation in the endoplasmic reticulum and the formation of cytoplasmic lipid droplets. We found that the number of the latter was related to the TrkB expression level. This result was confirmed thanks to the creation of a HEK cell line which over-expresses TrkB. We demonstrated that BDNF-induced TrkB activation leads to the formation of cytoplasmic lipid droplets, which can be abolished by K252a, an inhibitor of TrkB. So, MCARS microspectroscopy proved useful in characterizing cancer cells displaying an aberrant lipid metabolism.

## Introduction

Cancer is characterized by an uncontrolled cell growth, and tumor cell dissemination enabling the spread of cancer from its site of origin^1^. For a number of years, studies have focused on the identification of specific biomarkers associated with cancer processes. Biomarkers comprise many molecular structures such as DNA, mRNA, proteins or metabolites. However, in most cases, cancer biomarkers consist of proteins such as receptors at the cell surface^1,2^. Human epidermal growth factor receptor (ErbB) family has been the first receptor family discovered as involved in human cancers. In this family, the most common receptors are EGFR (Epithelial Growth Factor Receptor) and HER2 (Human Epidermal Growth Factor Receptor 2)^3^. Other biomarkers have also been discovered which are specific for certain types of cancer. For example, breast cancer can be detected by the presence of biomarkers such as HER2 or BRCA1, while KRAS and TP53 biomarkers are linked to non-small cell lung cancer^4^. Aside from distinguishing cancer cells from healthy ones, these biomarkers also allow to determine the stage of the cancer.

Neurotrophins have been discovered in the central and peripheral nervous systems, thereby regulating the growth and differentiation of neurons^5^. The neurotrophin family is composed of three major receptors, Trk A, B and C, associated with specific ligands: NGF binds to TrkA, BDNF and NT-4 bind to TrkB and NT-3 binds to TrkC^6^. When they are expressed in other human tissues, neurotrophins are associated with tumorigenesis, metastases, for example in ovarian, breast, and colorectal cancers^7^. The BDNF/TrkB complex is mainly correlated with colorectal cancer (CRC) and is of poor prognosis^8^. BDNF binding to TrkB induces an autophosphorylation of serine residues in the TrkB intracellular domain and triggers an activation of three signaling pathways: Ras/MAPK, PI3K/Akt and PLC-gamma/PKC, all of them leading to changes in cell metabolism and to cell proliferation^7^.

Our research work deals with the detection and identification of abnormal features of cancer cells thanks to multiplex coherent anti-Stokes Raman scattering (MCARS) microspectroscopy. MCARS microspectroscopy is a label-free and non-destructive imaging technique. It is based on a four-wave mixing process exploiting two laser beams, i.e. a pump beam at frequency ω_p_ and a Stokes beam at frequency ω_s_. When these two beams are tightly focused, an anti-Stokes beam is generated at frequency 2ω_p_ − ω_S_, corresponding to vibrational modes of the probed molecule. MCARS microspectroscopy is a highly sensitive technology that is able, in the mid-infrared spectral domain, to visualize proteins, lipids and heterochromatin inside the cells^9,10^.

Changes in lipid metabolism have been successfully addressed by vibrational spectroscopy and, more specifically, Raman spectroscopy, for example in cells expressing the HER2 receptor^11^, or in cells expressing EGFR^12^. The aim of our study was to use MCARS microspectroscopy in order to visualize and evaluate modifications of lipid metabolism induced by BDNF binding to the TrkB receptor in *TrkB*-transfected HEK cells. We correlated the lipid droplet content of the cells to the TrkB expression level in the three colorectal cancer cell lines, each one being representative of a different stage of CRC.

## Results

### Expression of the TrkB receptor and lipid droplet content of colorectal cancer cells

**Figure 1:**
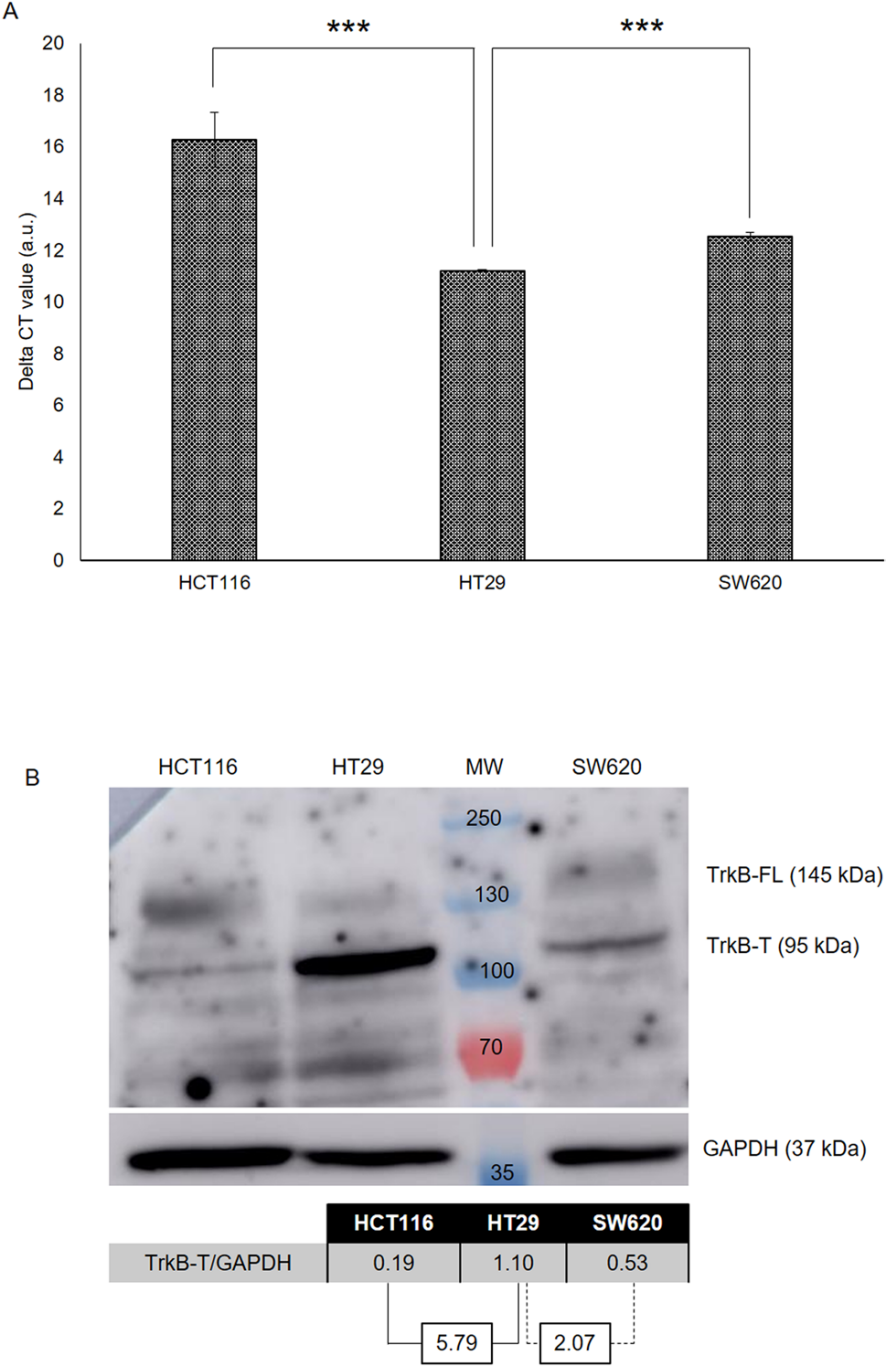
Expression of *TrkB* and TrkB in three CRC cell lines. (**A**) Expression of *TrkB* estimated by qRT-PCR from 2 ng of cDNA. Delta CT values were calculated using *GAPDH* expression as a reference (N = 2). (**B**) Western-blot analysis of TrkB expression. After protein extraction, a western-blot of TrkB was performed with 50 µg of extracted protein. For each cell line, relative expression of TrkB-T was calculated using GAPDH as a reference control (N = 2). MW: molecular weight, ***p value < 0.001. Full-length blots are presented in Supplementary Fig. 2.

The presence of the TrkB receptor was detected in three colorectal cancer cell lines representative of three different stages of the pathology: HCT116 (stage I), HT29 (stage II) and SW620 (stage III). Quantitative RT-PCR showed that these three cell lines expressed *TrkB* at different levels (Fig. 1A), with the highest expression level being found in HT29 (Delta CT: 11.2 ± 0.03). Expression of *TrkB* was 1.45 and 1.1 times lower in HCT116 and SW620, respectively. This observation was well in accordance with the levels of the corresponding proteins (Fig. 1B and Supplementary Fig. 2). The truncated form of the receptor (TrkB-T) was always more abundant than the full-length protein (TrkB-FL), whatever the colorectal cell line. This predominance has already been observed in the SW620 cell line^13^. The level of TrkB-T in HT29 was 5.79- and 2.07-fold higher than in HCT116 and SW620, respectively (Fig. 1B).

**Figure 2:**
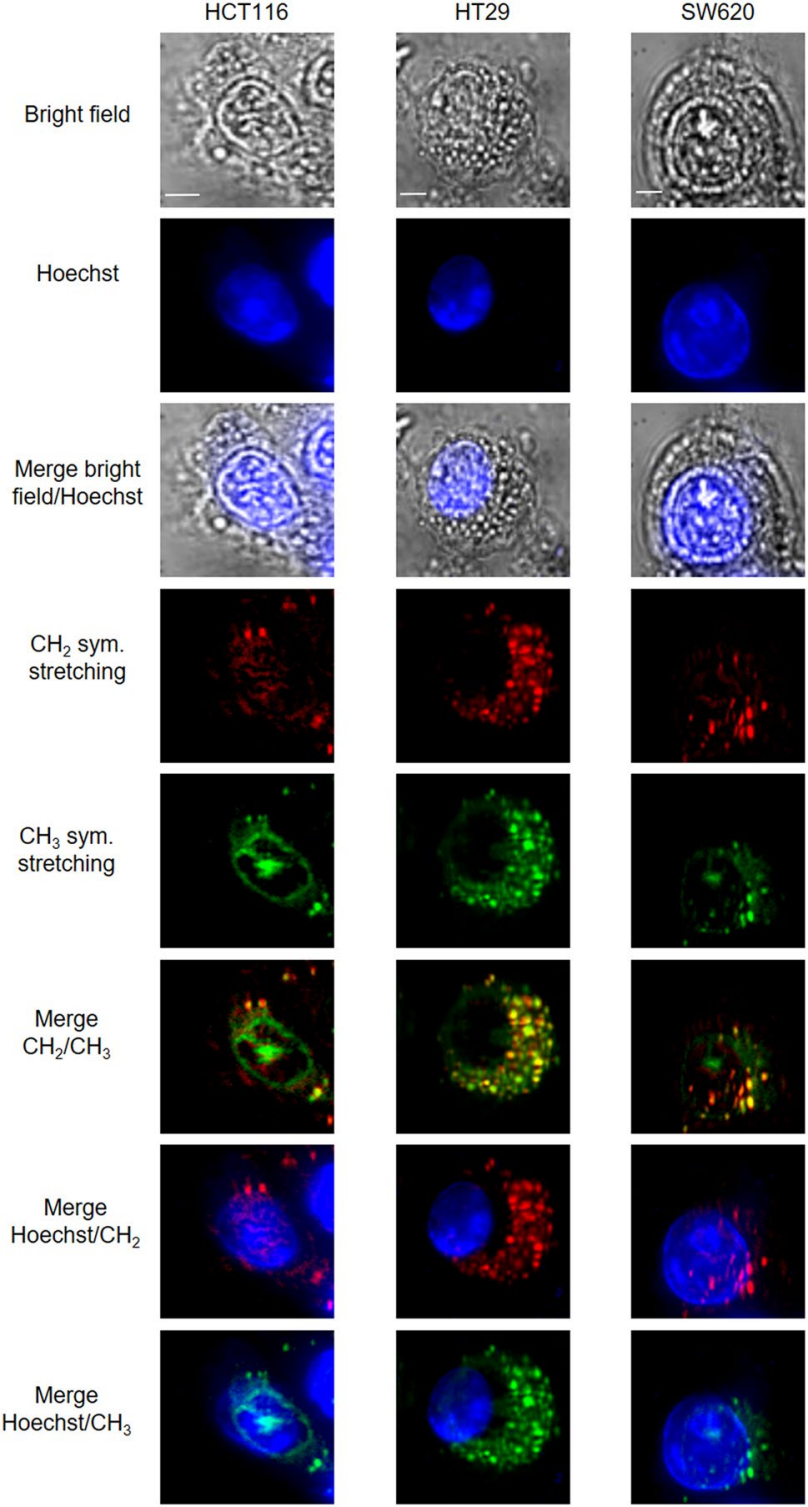
MCARS analysis of CRC cell lines. MCARS spectral images of living HCT116, HT29 and SW620 cells including bright-field, and fluorescence (Hoechst 33342) images. MCARS images were reconstructed from signal integration at 2850 cm^−1^ (CH_2_ symmetric stretching) and 2930 cm^−1^ (CH_3_ symmetric stretching) (N = 3). Scale bar, 5 µm.

These three cell lines have also been analyzed by MCARS microspectroscopy by mapping with a high spectral resolution (< 1 cm^−1^) in the 2500–3200 cm^−1^ spectral range. The CH_2_ (2850 cm^−1^, represented in red in Supplementary Fig. 3) and CH_3_ (2930 cm^−1^, represented in green in Supplementary Fig. 3) vibrational signatures are mainly associated with lipid and protein contents, respectively. MCARS cell images showed the presence of strong cytoplasmic signals in relation with the two vibrational signatures (Fig. 2). These two signatures overlapped and revealed an enrichment in lipids and proteins, leading to the formation of punctiform structures inside the cytoplasm of the three cell lines; the heaviest population was observed in HT29 cells (Supplementary Fig. 4). We observed the same type of punctiform structures when the cells were stained with BODIPY, a specific marker of neutral lipids, which constitute the main part of the lipid droplets (Supplementary Fig. 5). HT29 cells showed the strongest BODIPY staining, in comparison with HCT116 and SW620 cells. The BODIPY staining corresponds to the CARS labelling at 2850 cm^−1^, highlighting the CH_2_ bond associated with the lipid droplets. This observation method allows the visualization of lipid droplet similar in size, morphology and distribution in images obtained by fixative BODIPY staining^14^. We conclude that lipid metabolism is enhanced in the HT29 cell line in comparison with the two other ones.

### Analysis of stable HEK293 cell line overexpressing the TrkB receptor

**Figure 3:**
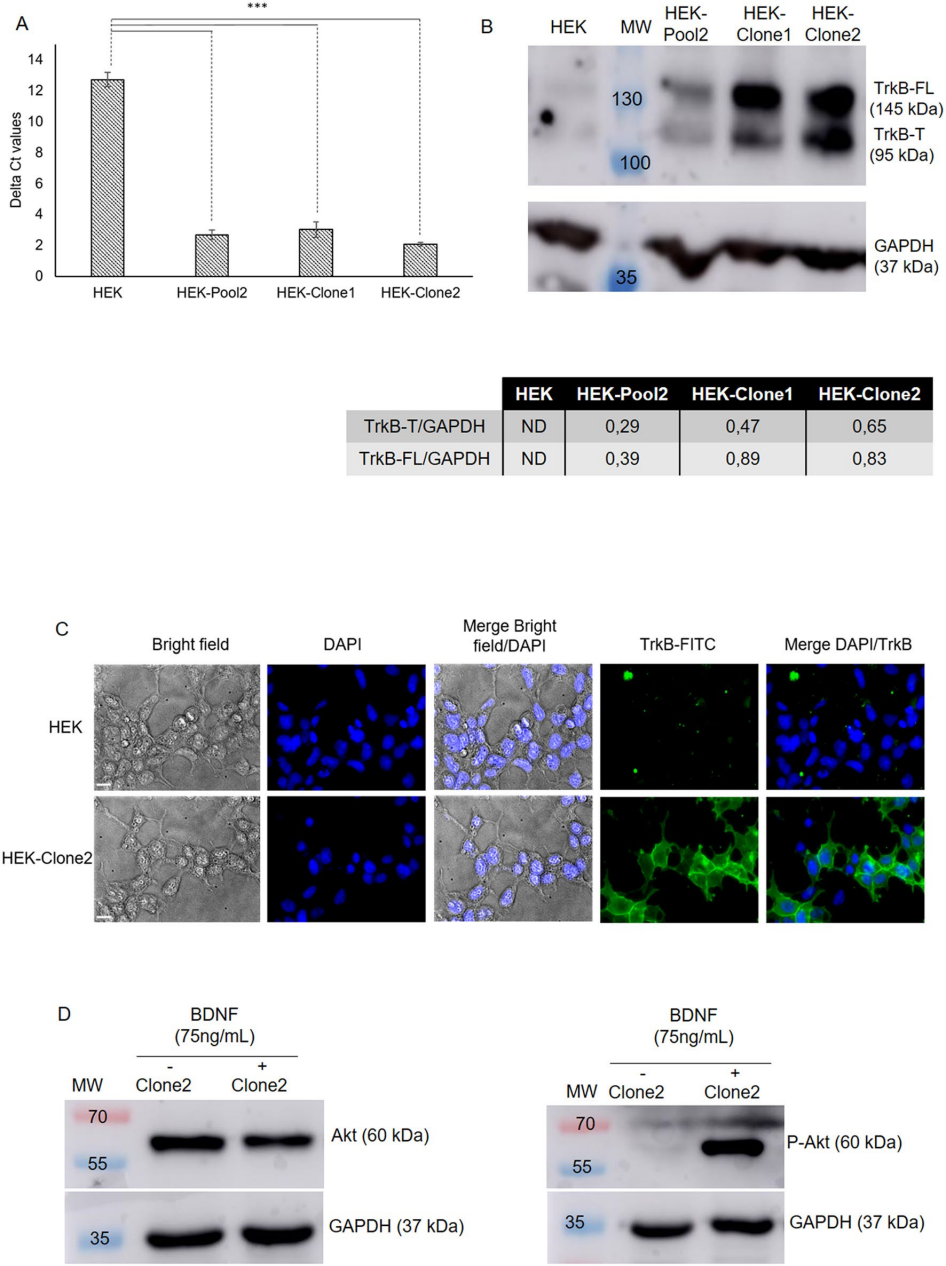
TrkB expression in transfected HEK cells. (**A**) *TrkB* expression was estimated by qRT-PCR from 2 ng of cDNA. Delta CT values for each condition was calculated using *GAPDH* expression as a reference (N = 5). (**B**) Western-blot analysis of TrkB expression in HEK clones. After protein extraction, western-blot of TrkB was performed with 50 µg of extracted protein. For each condition, relative expressions of TrkB-FL and TrkB-T were determined using GAPDH as a reference (N = 5). (**C**) Analysis of TrkB activation by immunofluorescence analysis of parent HEK and HEKClone2 cells. Binding of anti-hTrkB to the TrkB receptor was detected with a secondary antibody conjugated to Alexa Fluor 488 (green fluorescence). Nuclei were stained with DAPI (blue fluorescence) (N = 3). (**D**) HEK-Clone2 cells were treated with BDNF (75 ng/mL) during 1 h. Western-blots were realized on 50 µg of extracted protein; Akt and P-Akt (phospho-Akt) were detected on separate blots with the corresponding specific antibodies (N = 3). *ND* not Determined, *MW* molecular weight. ***p value < 0.001. Full-length blots presenting the TrkB-FL and TrkB-T are presented in Supplementary Fig. 6 and for Akt and P-Akt in Supplementary Fig. 7.

To ascertain that a link could exist between the expression level of the TrkB receptor its activation and the accumulation of lipid droplets in cells, the HEK293 cell line, which virtually does not express *TrkB,* was transfected with pCDNA3.1+/*TrkB*. Two transfected clones (clones 1 and 2) presented high expression levels of *TrkB* (Fig. 3A); *TrkB* was very poorly expressed in the parent HEK cells (CT value > 30). A slightly lower expression level was observed in HEKClone1 (Delta CT 3.01 ± 1.14) compared with HEK-Clone2 (Delta CT 2.07 ± 0.30). Western blot analysis confirmed this result; the total amount of TrkB in HEK-Clone2 was 1.1-fold higher than in HEK-Clone1. In both clones, the two forms of the TrkB receptor were present (Fig. 3B and Supplementary Fig. 6). HEK-Clone2 was chosen for further experiments.

### MCARS microspectroscopy analysis of BDNF-induced TrkB activation

Immunolabeling of the TrkB receptor revealed its location in the plasma membrane of the cells (Fig. 3C). To ensure that the TrkB receptor was functional, HEK-Clone2 cells were incubated with brain-derived neurotrophic factor (BDNF). Binding of BDNF to TrkB activates the PI3K/Akt signaling pathway, and induces the phosphorylation of Akt (P-Akt). This phosphorylated form is present only in BDNF-treated cells (Fig. 3D and Supplementary Fig. 7).

**Figure 4:**
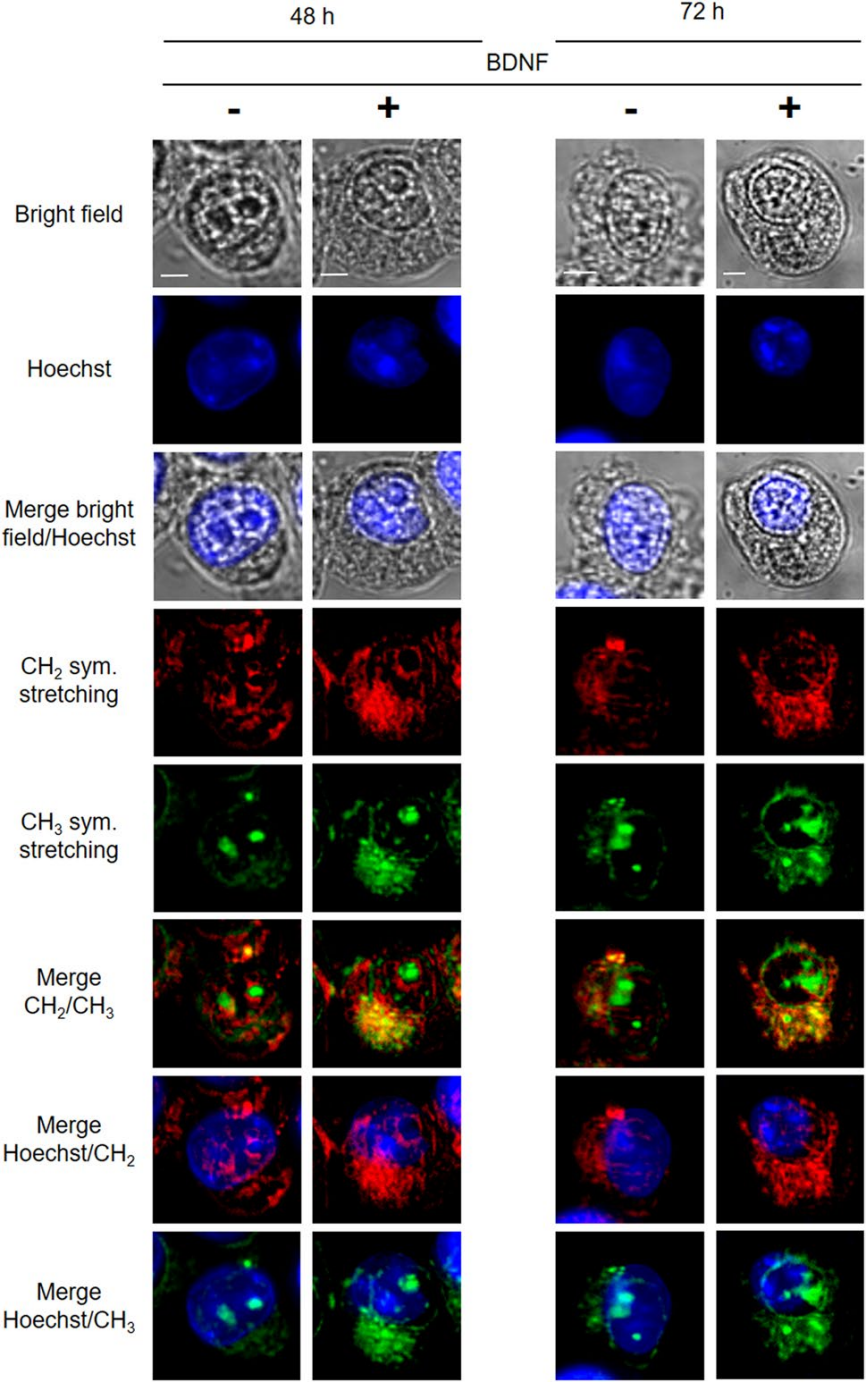
MCARS spectroscopy of HEK-Clone2 after BDNF-induced TrkB activation. HEK-Clone2 cells were incubated with, or without BDNF (75 ng/mL), during 48 and 72 h. From top to bottom: bright-field, fluorescence (Hoechst 33342), MCARS images reconstructed from signal integration at 2850 cm^−1^ (CH_2_ symmetric stretching) and 2930 cm^−1^ (CH_3_ symmetric stretching) (N = 5). Scale bar, 5 µm.

CH_2_ and CH_3_ vibrational signatures were mainly found in the cytoplasm of HEK-Clone2 cells, whatever the conditions (Fig. 4). These two spectral contributions slightly overlap, as in the case of colorectal cancer cell lines. The CH_2_ signatures were mainly located close to the nucleus, and correspond to ER^15^, and their intensities increased in BDNF-treated cells**.** Signal intensity increased with treatment times and, after 72 h, a few lipid droplets appeared (Fig. 4 and Supplementary Fig. 8). By contrast, the CH_3_ peak at 2930 cm^−1^ seems to be independent of cell treatment, which could be justified by its lower specificity (Supplementary Fig. 9). This signal includes the CARS signals of lipids, proteins and 5-methylcytosines of heterochromatin^15^. BODIPY staining revealed an accumulation of neutral lipids after 48 h of treatment, and a few lipid droplets appeared after 72 h, as shown with MCARS analysis (Supplementary Fig. 10).

In parallel, HEK cells, treated of not with BDNF during 72 h, were analyzed with MCARS microspectroscopy did not show any difference concerning the CH_2_ signal location (Supplementary Fig. 11). The same result was obtained after BODIPY staining which only revealed the endoplasmic reticulum (Supplementary Fig. 12).

**Figure 5:**
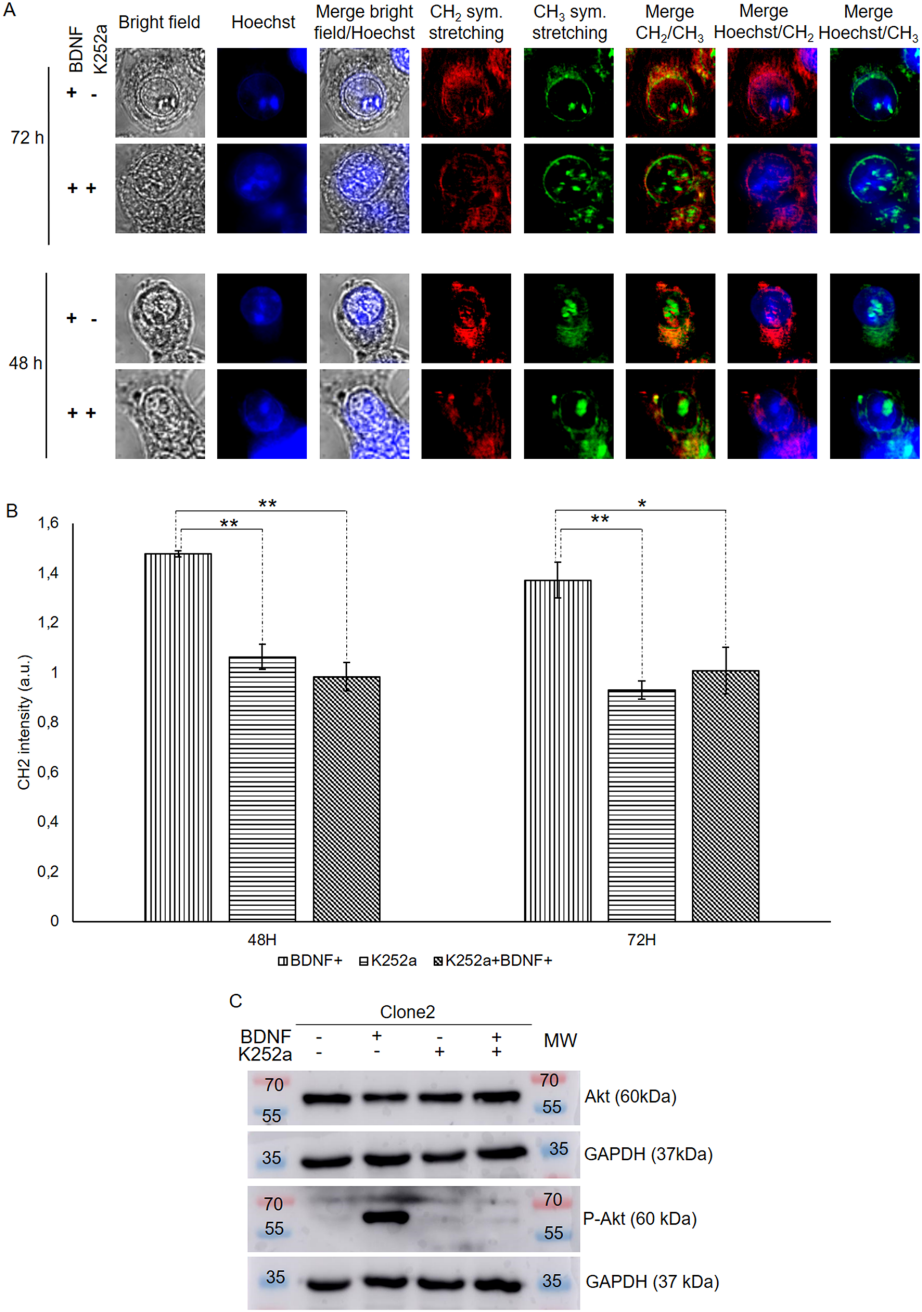
K252a inhibition of BDNF-induced TrkB activation. Analysis of HEK-Clone2 cells incubated with BDNF (75 ng/mL), and with or without K252a (100 nM) during 1 h. (**A**) MCARS microspectroscopy of HEK-Clone2 after a dual treatment. HEK-Clone2 cells were incubated with BDNF (75 ng/mL) with or without K252a (100 nM), during 48 and 72 h, the figure includes bright field and fluorescence (Hoechst 33342) images. MCARS images were reconstructed from signal integration at 2850 cm^−1^ (CH_2_ symmetric stretching) and 2930 cm^−1^ (CH_3_ symmetric stretching). Scale bar, 5 µm. (**B**) Quantification of the CH_2_ signal intensity in HEK-Clone2 cells by MCARS analysis after a dual treatment for 48 and 72 h. We used HEK-Clone2 with no BDNF treatment as reference. (**C**) Western-blot of Akt and P-Akt (phospho-Akt) realized with 50 µg of extracted protein. Full-length blots for Akt and P-Akt are presented in Supplementary Fig. 7. *p < 0.05; **p < 0.01. For all experiments N was at least equal to 3.

In order to demonstrate that lipid accumulation in ER and lipid droplet formation strictly depend on TrkB activation, we treated HEK-Clone2 and HT29 cells with K252a, an inhibitor of the neurotrophin receptor family^16–19^. When HEK-Clone2 cells were simultaneously incubated with BDNF and K252a, MCARS microspectroscopy analysis revealed an absence of lipid droplets formation (Fig. 5A and Supplementary Fig. 13). A quantification of CH_2_ intensity for each condition was carried out, with the untreated HEK-Clone2 as a reference, either at 48 or 72 h. Following treatment with BDNF, we observed an increase in CH_2_ signal intensity after 48 and 72 h (1.47 ± 0.013 and 1.37 ± 0.073, respectively) compared with untreated HEK-Clone2 cells (signal intensity = 1). This increase was abolished when cells were treated either with K252a alone (1.06 ± 0.051 at 48 h and 0.93 ± 0.038 at 72 h) or with both K252a and BDNF (0.98 ± 0.056 at 48 h and 1.01 ± 0.093 at 72 h). The CH_2_ intensities were similar to those observed with control cells (Fig. 5B). Examination of BODIPY-stained cells led to the same conclusion. Lipid accumulation disappeared when HEK-Clone2 cells were treated with the TrkB antagonist K252a (Supplementary Fig. 14).

When HEK Clone2 cells were simultaneously incubated with BDNF and K252a, phosphorylation of Akt did not occur (Fig. 5C). The K252a antagonist has been shown to block the phosphorylation of the TrkB receptor and thus to block activation of the PI3Kinase signaling pathway^19^.

**Figure 6:**
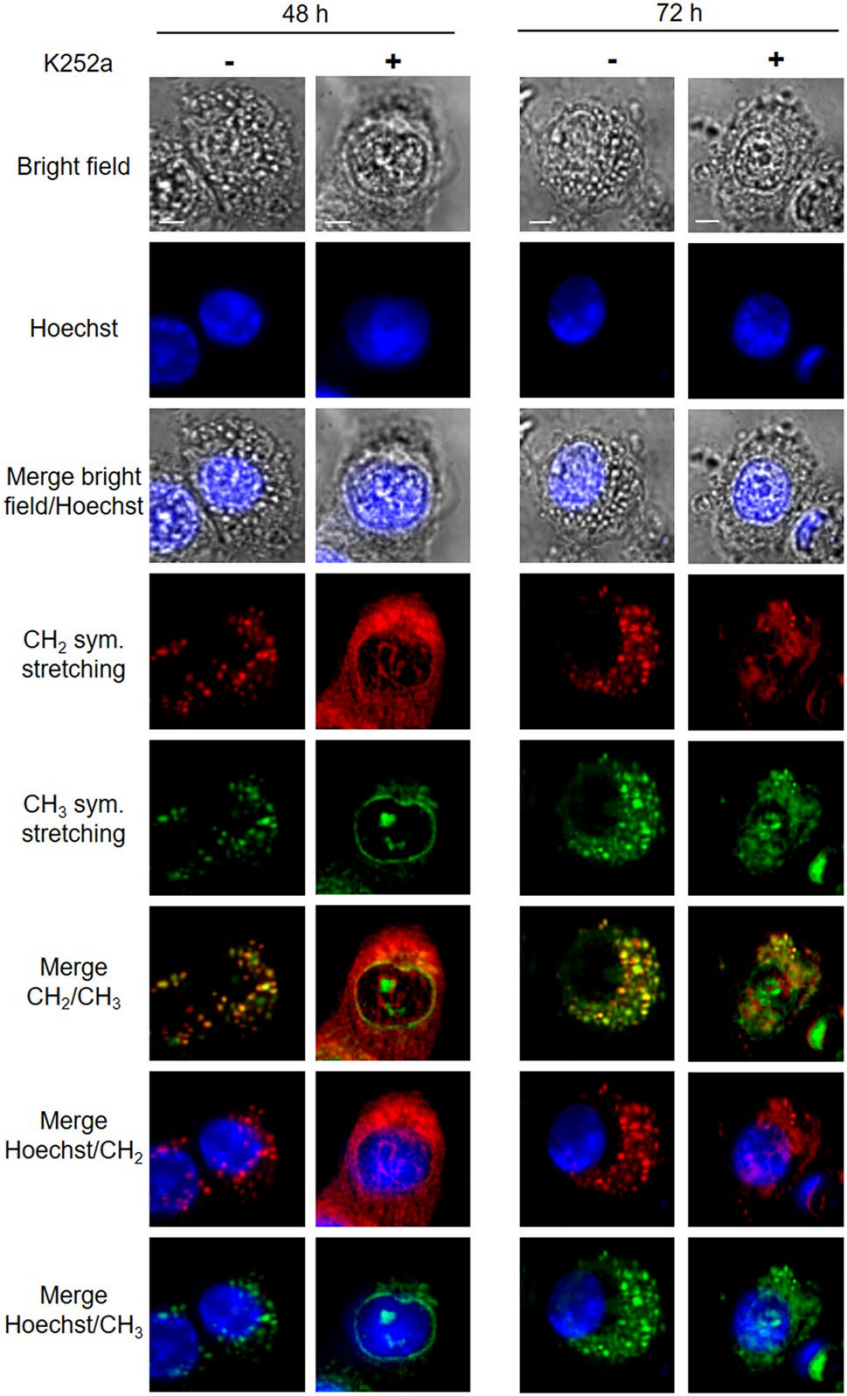
MCARS spectroscopy of HT29 cells incubated with K252a. HT29 cell lines were treated with K252a (100 nM), during 48 and 72 h. From top to bottom: bright-field, fluorescence (Hoechst 33342) images, MCARS images reconstructed from signal integration at 2850 cm^−1^ (CH_2_ symmetric stretching) and 2930 cm^−1^ (CH_3_ symmetric stretching), merged images (N = 2). Scale bar, 5 µm.

K252a also inactivated TrkB in HT29 cells. MCARS microspectroscopy of K252a-treated HT29 cells revealed a change in lipid organization; although lipids remained present in large quantities in the cytoplasm, they did not give rise to lipid droplets after 48 h of treatment, and only a small number could be seen after 72 h (Fig. 6 and Supplementary Fig. 15). This observation agreed with BODIPY staining (Supplementary Fig. 16). Both analyses showed that activation of the TrkB receptor is involved in the accumulation of neutral lipids, and so in the generation of lipid droplets.

## Discussion

A large number of cancer cells present an enhanced lipid metabolism^20^, which is frequently associated with a resistance to chemotherapy. This increase in lipid metabolism leads to neutral lipid accumulation in ER and to the formation of lipid droplets^21^. Lipid accumulation in cells can be visualized by MCARS microspectroscopy analysis, using the strong CH_2_ signature, which is mainly associated with lipid droplets^22,23^. Studies using vibrational microspectroscopy (Raman and CARS) have pointed out high lipid levels in cancer cells e.g. breast^24^, or colorectal^25^ cancers, as well as in circulating tumor cells^26,27^ that could be detected using the Stimulated Raman Scattering imaging flow cytometer, a marker-free detection technology^28^. However, none of these studies focused on the origin of this increased lipid content.

We thought that a receptor involved in colorectal cancer^7^ was a possible cause of this metabolic change. Accordingly, we performed MCARS microspectroscopy analyses of three colorectal cancer lines (HCT116, HT29, SW620), which expressed the neurotrophin receptor TrkB at different levels. We correlated the lipid droplet content of these cells with the expression level of TrkB and with its activation. A higher amount of lipid droplets was observed in cytoplasm of early cancer stage cells (HT29), as also observed in different breast cancer cell lines^29^. Nevertheless, these cell lines express other receptors, such as EGFR and HER2, that could also influence lipid metabolism^30^. So, in order to evaluate the influence of TrkB on lipid metabolism, we generated, from the HEK cell line, which does not express TrkB, several transfected clones that expressed TrkB. Cell treatment with BDNF induces an activation of one of the three main signaling pathways: the PI3Kinase/Akt pathway^6^. The study of these cells by MCARS microspectroscopy allowed us to observe a higher intensity of the CH_2_ signature in HEK-Clone2 treated cells. This CH_2_ signature was more intense and structured in cytoplasm at 48 h compared with untreated cells and, after 72 h of treatment, a few lipid droplets appeared in the cytoplasm. This observation was confirmed by cell staining with BODIPY, a dye which binds specifically to neutral lipids^31^. Klapper et al. showed that the lipid droplets labelling with BODIPY corresponded to the punctiform staining observed with CARS and Raman at 2845 cm^−1^, through co-localization method. They clearly concluded that vibrational microscopy, in high wavenumbers, allows the observation of lipid droplets whose characteristics (size, morphology, and distribution) are similar to those observed with the fixative BODIPY staining method^14^.

Raman spectroscopy analysis of prostatic cancer cells showed that an activation of Pi3Kinase/Akt induces an accumulation of cholesteryl-ester, which contributes to the formation of lipid droplets^32^. We postulate that activation of TrkB induces the activation of the Akt pathway and leads to lipid droplet accumulation in cells.

To ensure that lipid droplet formation resulted from TrkB activation we used K252a, a specific inhibitor of tyrosine kinase receptors and more especially of neurotrophins^16,17^. When we analyzed HEK cells expressing TrkB simultaneously treated with BDNF and K252a, we observed an absence of the P-Akt form as well as no accumulation of lipids. Similar results were obtained when HT29 cells, which produce endogenous BDNF^33^, were treated with K252a.

Earlier work using lipid-specific dyes or immunolabeling of lipid droplets has shown that the biogenesis of lipid droplets proceeds from two major steps: accumulation of neutral lipids in ER bilayers^34^ followed by the formation of lipid droplets^35^. Thanks to MCARS microspectroscopy, we have been able—without prior staining—to differentiate these two steps in BDNF-treated HEK-Clone2 cells: accumulation of neutral lipids in ER which leads to a diffuse signal close to the nuclear envelope, as observed after 48 h of treatment, followed by the generation of lipid droplets leading to punctiform signals in cytoplasm after 72 h of treatment; we observed the same evolution in HT29 cells, which strongly express TrkB.

Accumulation of lipids, mainly in the form of droplets, can be considered as an additional hallmark of cancer^20^. This lipid increased has already been described in cancer tissues by Raman and CARS microspectroscopies, for example in hepatocellular^36,37^ and breast cancers^38^, as well as in circulating tumor cells (following a murine xenograft)^26,27^. However, these studies used either Raman or CARS microspectroscopies, two techniques which are hampered by their associated drawbacks: the former has a low sensitivity and presents the disadvantage of a low signal/noise ratio, while the latter requires a heavy processing algorithm (MCR-ALS), which is difficult to use, due to its complexity and to the long data processing time. In our study, we showed the possibility of a rapid and simple characterization of cancer cells according to their lipid composition, thanks to an affordable algorithm (Method of Maximum Entropy). Thus, lipid droplet formation is associated with tumor progression but also with the cancer stage. In fact, Geng et al. have determined a significantly higher quantity of droplets in high-grade glioblastoma and colorectal cancer compared with low-grade cancers and normal tissues, showing that the presence of cytoplasmic lipid droplets might constitute a diagnostic biomarker of cancer^39^.

Indeed, it has already been shown that lipid metabolism plays a significant role in cancer processes such as proliferation, resistance to chemotherapy and so on. Enhanced lipid metabolism is often associated with poor prognosis^40^. Moreover, it has also been demonstrated that a resistance to 5-fluorouracil and doxorubicin is correlated with an increase in the formation of lipid droplets in hepatocellular carcinoma and colorectal cancer cells^41^. Due to their hydrophobic core, able to attract and trap lipophilic drugs, the lipid droplets contribute to the chemoresistance ability of the cells as reported in the case of curcumin^42^. Drugs such as erlotinib, which possess an alkyne group can be specifically identified by CARS microspectroscopy in the silencing region of the Raman spectrum and localized in cells^43^. This kind of analysis could be extended to other drugs naturally bearing an alkyne group or to alkyne-modified drugs in order to determine their location in cells and to possibly attribute the chemoresistance to drug accumulation in a specific cell compartment.

So, MCARS microspectroscopy combined with a simple data processing could be a useful tool to: (1) identify cancer cells, (2) evaluate the tumor cell evolution and (3) predict their chemoresistance via the determination of their cytoplasmic lipid droplet content.

## Materials and methods

### Cell culture

HEK293 cells were cultured in Dulbecco’s modified Eagle’s medium (DMEM; Gibco, Life Technologies) supplemented with 10% fetal calf serum (Eurobio), 100 units/mL penicillin and 100 µg/mL streptomycin at 37 °C under a humidified atmosphere with 5% CO_2_.

Three colorectal cancer cell lines were used: HCT116 (stage I), HT29 (stage II) and SW620 (stage III). HCT116 and SW620 cell lines were cultured in RMPI 1640 (Gibco, Life Technologies) and HT29 cell line in DMEM media. Both media were supplemented with 10% fetal calf serum (Eurobio), 100 units/mL penicillin and 100 µg/mL streptomycin at 37 °C under humidified atmosphere and 5% CO_2_.

### Generation of the expression vector allowing the overexpression of the TrkB receptor

A DNA fragment encoding the coding sequence of *TrkB* was amplified by PCR using pCMV-XL4-*TrkB* as template and the following oligonucleotide primers: Forward primer: 5′ **GGATCC**AAGAGAGCCGCAAGCGC 3′ and Reverse primer: 5′ **TCTAGA**GCAGCTTGGTGGCCTCC 3′ (nucleotides in bold indicate the location of a BamHI site, and the antisense strand the location of a XbaI site). PCR conditions were as followed: Denaturation (98 °C-30 s), 35 cycles of denaturation (98 °C-10 s), hybridization (63 °C-20 s) and extension (72 °C-45 s). The PCR was achieved with a final extension (72 °C-5 min) using Phusion High Fidelity DNA Polymerase (ThermoScientifique). PCR product was introduced into clone vector pGEM-Teasy (Promega). A selection was then performed to find clones with pGEM-Teasy containing the *TrkB* sequence (pGEM-Teasy-*TrkB*).

The vector pCDNA3.1+ was treated with calf intestinal alkaline phosphatase after being digested with BamH1 and XbaI, and ligated with a *TrkB* DNA fragment that has been obtained from clone vector pGEM-Teasy-*TrkB*. A selection was performed to obtain a clone with vector containing the *TrkB* sequence called pCDNA3.1+/*TrkB*. The *TrkB* sequence in pCDNA3.1+ was confirmed by DNA sequencing.

### Generation of TrkB overexpression in HEK cell line

HEK cells were transfected with pCDNA3.1+/*TrkB* using JetPei (PolyPlus transfection). Briefly, Solution A with 2 µg of pCDNA3.1(+)/*TrkB* in 50 µL of NaCl (150 mM) and solution B with 4 µL of JetPei with 50 µL of NaCl (150 mM) were prepared. After 5 min at room temperature, both solutions were mixed and incubated 20 min at room temperature. The mix was then put on cells. After 24 h at 37 °C and 5% CO_2_, the transfection media was replaced by complete media with 750 µg/mL Geneticin (G418-Roth) as selection marker. Several clones were selected and characterized by qRT-PCR giving us their *TrkB* expression level.

### Quantitative real-time PCR

Total RNA was extracted using the RNeasy minikit (Qiagen). Then it has been quantified by measuring the absorbance at 260 nm using the Nanodrop spectrophotometer ND-1000 (NanoDrop Technologies). A high-capacity cDNA reverse transcription kit (Applied Biosystems) was used for the conversion of 10 µg of total RNA to single-stranded cDNA suitable for quantitative PCR applications. Quantitative PCR was performed from 2 ng total cDNA in an ABI Prism 7900 Sequence Detector System (Thermo Fisher Scientific) using 40 cycles at 95 °C for 15 s followed by 60 °C for 1 min. Taqman primers and probe sets used in this study were as follows: *TrkB* (NTRK2) (Hs00178811_m1), *GAPDH* (Hs02786624_g1). Gene expression data were collected using the SDS software, version 2.2.2, (Applied Biosystems). The gene expression comparison has been carried out using delta-CT, corresponding to the CT (cycle threshold) of the gene of interest relative to *GAPDH* as a reference control.

### Western blot analysis

Proteins were extracted from cells with RIPA lysis buffer [50 mM Tris–HCl, pH 8, 150 mM NaCl, 0.5% sodium deoxycholate (*w/v*), 1% NP-40, 0.1% SDS (*v/v*) and protease inhibitor cocktail (complete; Roche Diagnostics)]. Protein extract was centrifuged at 12,000*g* for 30 min at 4 °C. Supernatants were recovered and the protein concentration was estimated using a Bicinchoninic acid (BCA) protein assay (Sigma-Aldrich) with Bovine Serum Albumin as a standard.

Fifty micrograms of extracted proteins were separated under denaturing and reducing conditions with SDS polyacrylamide gel [8–10% (*w/v*)] and then transferred to Hybond C-extra nitrocellulose membrane (GE Healthcare). Membranes were then blocked in 5% nonfat dried milk (*w/v*) in TBST (50 mM Tris–HCl, 150 mM NaCl, 0.1% Tween-20, pH 7.4) during 1 h at room temperature and then incubated overnight at 4 °C with specific primary antibodies diluted in 5% nonfat dried milk (*w/v*) in TBST at: 1:150 dilution of anti-hTrkB (R&D systems, mab3971), 1:250 dilution of anti-Akt and anti-phospho Akt (R&D systems, MAB2055 and AF887 respectively) and 1:2000 dilution of anti-GAPDH (R&D systems, AF5718). After three washings in TBST, membranes were incubated for 1 h at room temperature with 1:1000 dilution of secondary HRP conjugate antibodies (Dako) in 1% non-fat dried milk (*w/v*) in TBST. After last three washings, immunoblots were developed using BM Chemiluminescence Western-blotting substrate (peroxidase, POD; Applied Science). Analysis of spectral band intensities and chemical image generation were carried out using ImageJ software (NIH, v6) for relative quantification purposes.

### Immunofluorescence analysis

HEK and HEK-Clone2 were seeded at 7 × 10^4^ cells per well (Lab-Tek, Scientific). After 2 days, cells were washed three times with PBS and fixed with 4% of paraformaldehyde in PBS (*v/v*) for 10 min. Then cells were saturated for 1 h at room temperature using PBS-BSA 5%. Cells were then incubated with primary antibodies: anti-hTrkB in PBS-BSA 1% during overnight at 4 °C. After three washes in 0.1% Tween20-PBS, cells were incubated for 1 h at room temperature with polyclonal Alexa Fluor-conjugated secondary antibodies (Alexa Fluo 488, Life Technologies). After three washes with 0.1% Tween20-PBS, nuclei were stained using 1 µg/mL DAPI (Sigma-Aldrich). Cells were washed three times before being mounted on slides with Mowiol 4–88 mounting medium and sealed with nail polish.

Stained cells were observed with an epifluorescence microscope (Leica DMI4000B). Data were processed using MetaMorph software (Molecular Devices).

### BDNF and K252a treatments

Different conditions were used according to the analyses performed.

#### For proteins extraction

HEK293 and HEK-Clone2 cells were seeded at a density of 10^4^ cells/cm^2^ and cultured 2 days before treatment with 75 ng/mL recombinant human BDNF (Peprotech)^44^ or with 100 nM of K252a (Alomone)^45^ Cells were harvested after 1 h of treatment and proteins were extracted. A dual treatment was performed with BDNF and K252a in order to block the TrkB activation.

#### For CARS analysis

HEK293 and HEK-Clone2 cells were seeded at a density of 35,000 cells per well on 18 mm diameter uncoated round glass coverslips and cultured 2 days before treatment with 75 ng/mL BDNF or with BDNF and K252a (100 nM). After 48 h and 72 h of treatment, cells were washed three times with PBS and nuclei were labelled with 10 µg/mL Hoechst 33342 (Thermo Fisher Scientific) during 15 min. Cells were then washed, and the coverslip sealed with nail polish on a microscopy glass slide.

### MCARS microspectroscopy

The spectroscopic setup used in this work is presented in^15^. The lateral, axial and spectral resolutions of the CARS microspectroscope were ~ 300 nm, 2 µm, and 0.8 cm^−1^, respectively. During all experiments, the laser powers of pump and Stokes radiations at the sample position were set at 55 mW and 9 mW, respectively. At this laser power (64 mW in total), no morphological change of cells was observed. This was confirmed by visualizing the sample with bright field and/or fluorescence imaging.

Spectra were acquired from 2500 to 3200 cm^−1^ with 50 ms pixel dwell time and processed by using the maximum entropy method (MEM) so as to extract the pure vibrationally resonant signal, which corresponds to the imaginary part of the third order nonlinear susceptibility (Im{χ^(3)^})^46^. CARS chemical images were reconstructed for two Raman modes: CH_2_ symmetric stretching, associated to lipids, at 2850 cm^−1^ and CH_3_ symmetric stretching, associated to proteins, at 2930 cm^−1^.

Quantification of the CH_2_ signature, at 2850 cm^−1^, was realized in all mapping for all conditions and we used the ImageJ software (NIH, v6).

## Supplementary information

Supplementary Information.

## Data Availability

The datasets generated during the current study are available from the corresponding author on reasonable request.
