# Supplementary material for: Multiplex coherent anti-Stokes Raman scattering microspectroscopy detection of lipid droplets in cancer cells expressing TrkB
Source: Sci Rep. 2020 Oct 7;10:16749. doi: 10.1038/s41598-020-74021-z (PMC7542145; doi:10.1038/s41598-020-74021-z)
Supplement: Supplementary file 1 — Supplementary Information. [file 41598_2020_74021_MOESM1_ESM.docx]

**Supplementary file for**

**Multiplex Coherent Anti-Stokes Raman Scattering microspectroscopy detection of lipid droplets in cancer cells expressing TrkB**

Tiffany Guerenne-Del Ben,^1^, Vincent Couderc^2^, Ludovic Duponchel^3^, Vincent Sol^1^, Philippe Leproux^2,4^, and Jean-Michel Petit^1*^

Supplementary file includes:

1. **Supplementary Methods**
2. **Supplementary figures**

**Supplementary Fig. 1 Analysis of lipid content following addition of fatty acids in cell culture medium.**

**Supplementary Fig. 2 Full-length of the blots presented in Fig. 1.**

**Supplementary Fig. 3 Mean of standard deviation of the vibrationally resonant CARS signal between 2500 and 3200 cm^-1^.**

**Supplementary Fig. 4 MCARS microspectroscopy of colorectal cancer cell lines, HCT116, HT29 and SW620.**

**Supplementary Fig. 5 BODIPY staining of colorectal cancer cell lines, HCT116, HT29 and SW620.**

**Supplementary Fig. 6 Full-length of the blots presented in Fig. 3B.**

**Supplementary Fig. 7 Full-length of the blots presented in Fig. 3D.**

**Supplementary Fig. 8 MCARS microspectroscopy of HEK-Clone2 after BDNF-induced TrkB activation.**

**Supplementary Fig. 9 Mean of standard deviation of the vibrational resonant CARS signal between 2500 and 3200 cm^1^.**

**Supplementary Fig. 10 BODIPY staining of HEK-Clone2 cells after BDNF-induced activation of the TrkB receptor**.

**Supplementary Fig. 11 MCARS microspectroscopy of HEK after BDNF-induced TrkB activation.**

**Supplementary Fig. 12 BODIPY staining of HEK cells after BDNF-induced activation of the TrkB receptor.**

**Supplementary Fig. 13 MCARS microspectroscopy of HEK-Clone2 after K252a inhibition of BDNF-induced TrkB activation.**

**Supplementary Fig. 14 BODIPY staining of HEK-Clone2 after K252a inhibition of BDNF-induced TrkB activation.**

**Supplementary Fig. 15 MCARS microspectroscopy of HT29 cell line after K252a inhibition.**

**Supplementary Fig. 16 BODIPY staining of HT29 cell line treated with K252a**

1. **References**
2. **Supplementary Methods**

**Lipid droplet staining with BODIPY**

*Fatty acids treatment*

To validate BODIPY ability to label lipid droplets, a treatment with fatty acids, which stimulates the neutral lipid synthesis and thus forms lipid droplets, has been carried out (Supplementary Fig. 1). For this reason, HEK cells were seeded at a density of 10^4^ cells per cm^2^ in 12-well plate and cultured in DMEM medium supplemented with 10% FBS, 100 units/mL penicillin and 100 µg/mL streptomycin at 37 °C and 5 % CO_2_. After two days, cells were treated with oleic acid at 0.7 mM (Sigma-Aldrich) during 24 h. Then, cells were washed three times with PBS and fixed with PFA (4 % (*v/v*) in PBS) for 10 min. After three washings with PBS, cells were stained with 5 µg/mL BODIPY (Thermofisher) for 1 hour^1^. After three washes, nuclei were stained using 1 µg/mL with DAPI. Cells were rinsed three times before being mounted on slides with Mowiol 4-88 mounting medium and sealed with nail polish.

Concerning HEK and HEK-Clone2 cells treated with BDNF and K252a, or without K252a or without BDNF during 48 h and 72 h, the same protocol was used.

1. **Supplementary figures**

**
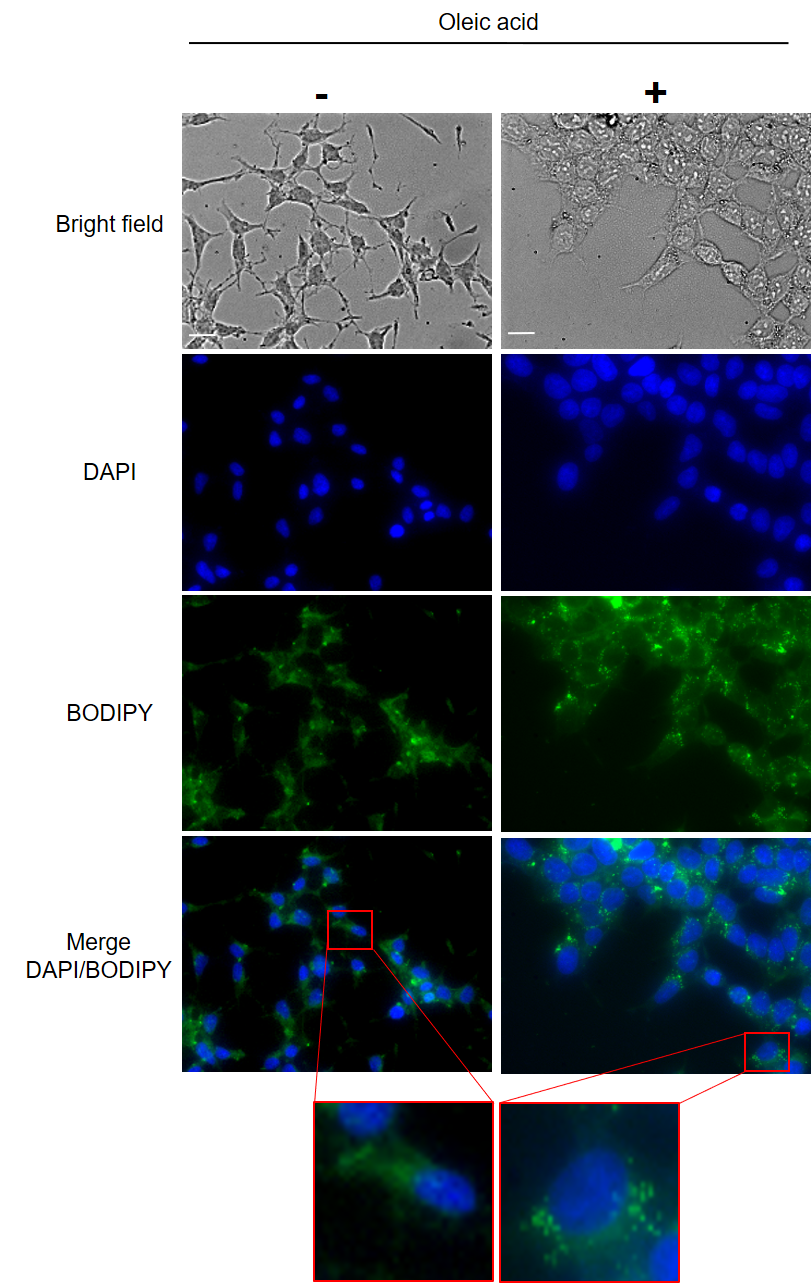
**

**Supplementary Fig. 1 Analysis of lipid content following addition of fatty acids in cell culture medium.** HEK cells were treated with, or without oleic acid at 0.7 mM, for 24 hours. Then cells were fixed using PFA and stained with BODIPY 493/503 (green fluorescence) for lipid and DAPI (blue fluorescence) for nuclei. Scale bar, 10 µm.


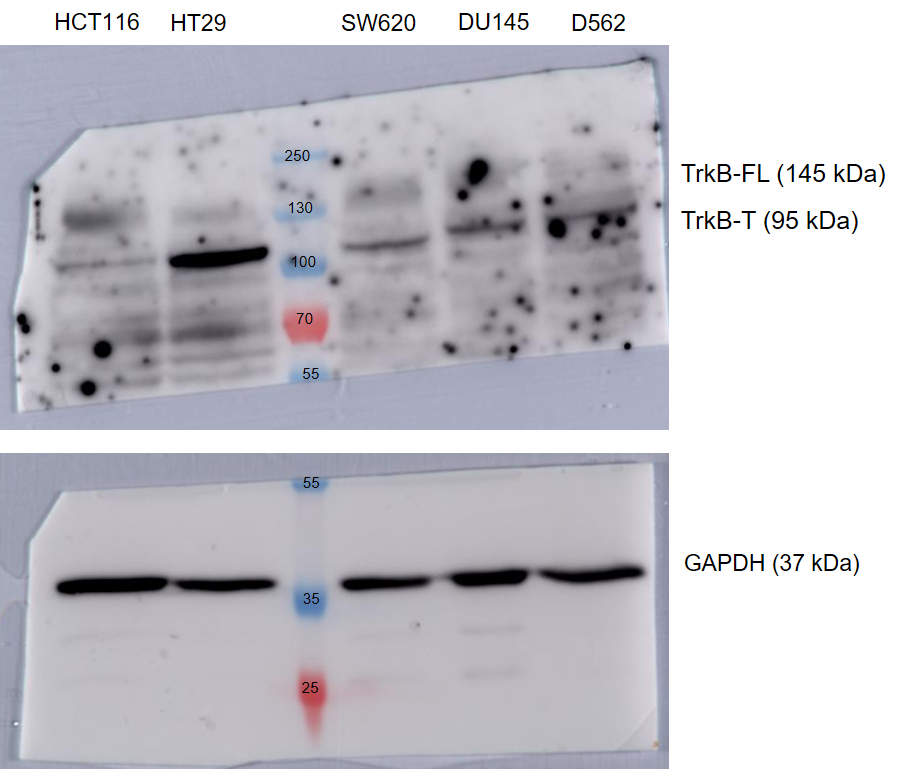


**Supplementary Fig. 2 Full-length of the blots presented in Fig. 1.** Western-blot analysis of TrkB expression in three colorectal cancer cell lines (HCT116, HT29, and SW620), prostate cancer cell line (DU145) and hypopharyngeal squamous carcinoma cell line (D562). After protein extraction, a western-blot of TrkB was performed with 50 µg of extracted protein.

**
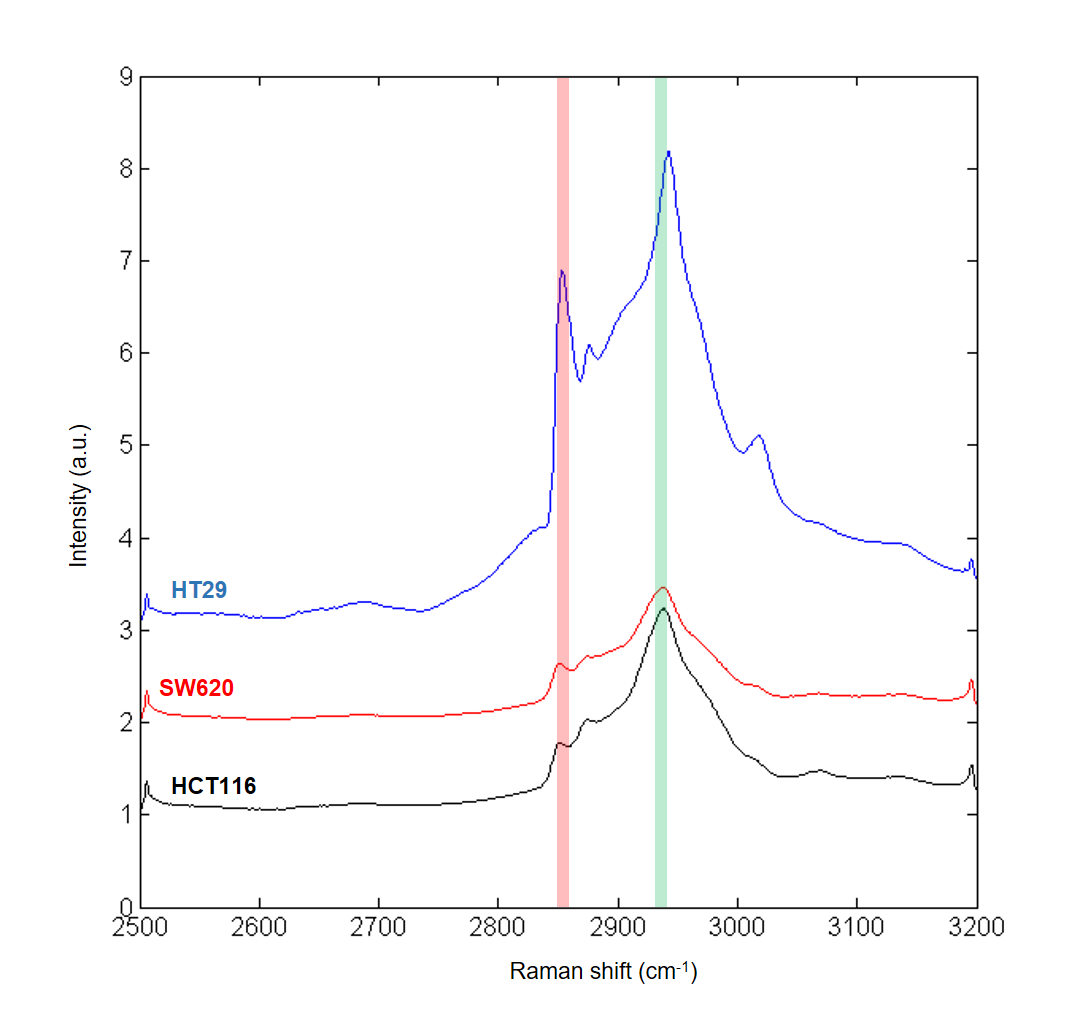
**

**Supplementary Fig. 3 Mean of standard deviation of the vibrationally resonant CARS signal between 2500 and 3200 cm^-1^.** computed over the whole region of interest for 8 cells for HCT116, 11 cells for HT29 and 6 cells for SW620. CH_2_ and CH_3_ spectral contributions are highlighted in red and green, respectively.


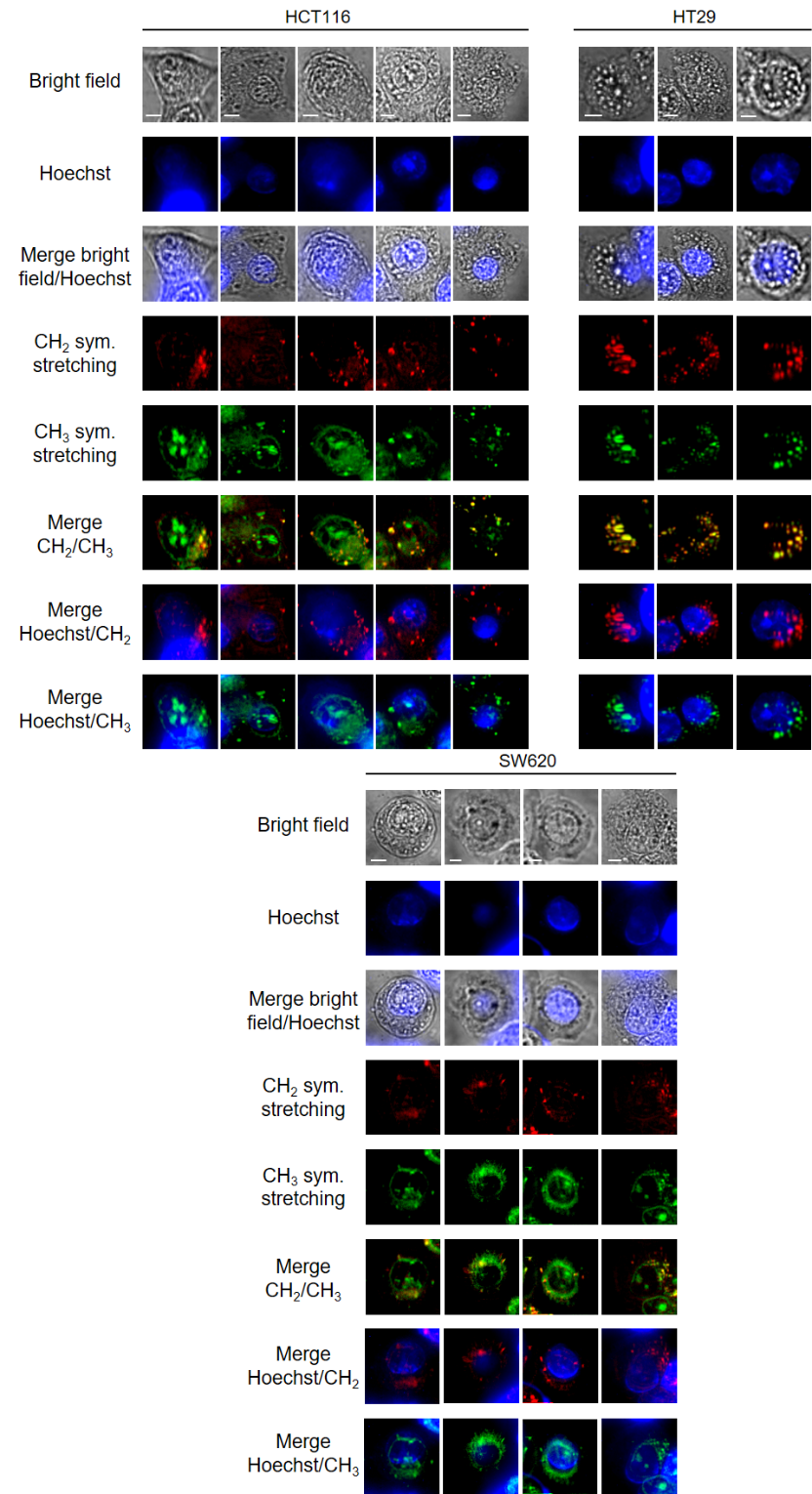


**Supplementary Fig. 4 MCARS microspectroscopy of colorectal cancer cell lines, HCT116, HT29 and SW620.** MCARS spectral images of living HCT116, HT29 and SW620 cells including bright-field, and fluorescence (Hoechst 33342) images. MCARS images were reconstructed from signal integration at 2850 cm^-1^ (CH_2_ symmetric stretching) and 2930 cm^-1^ (CH_3_ symmetric stretching). Scale bar, 5 µm.


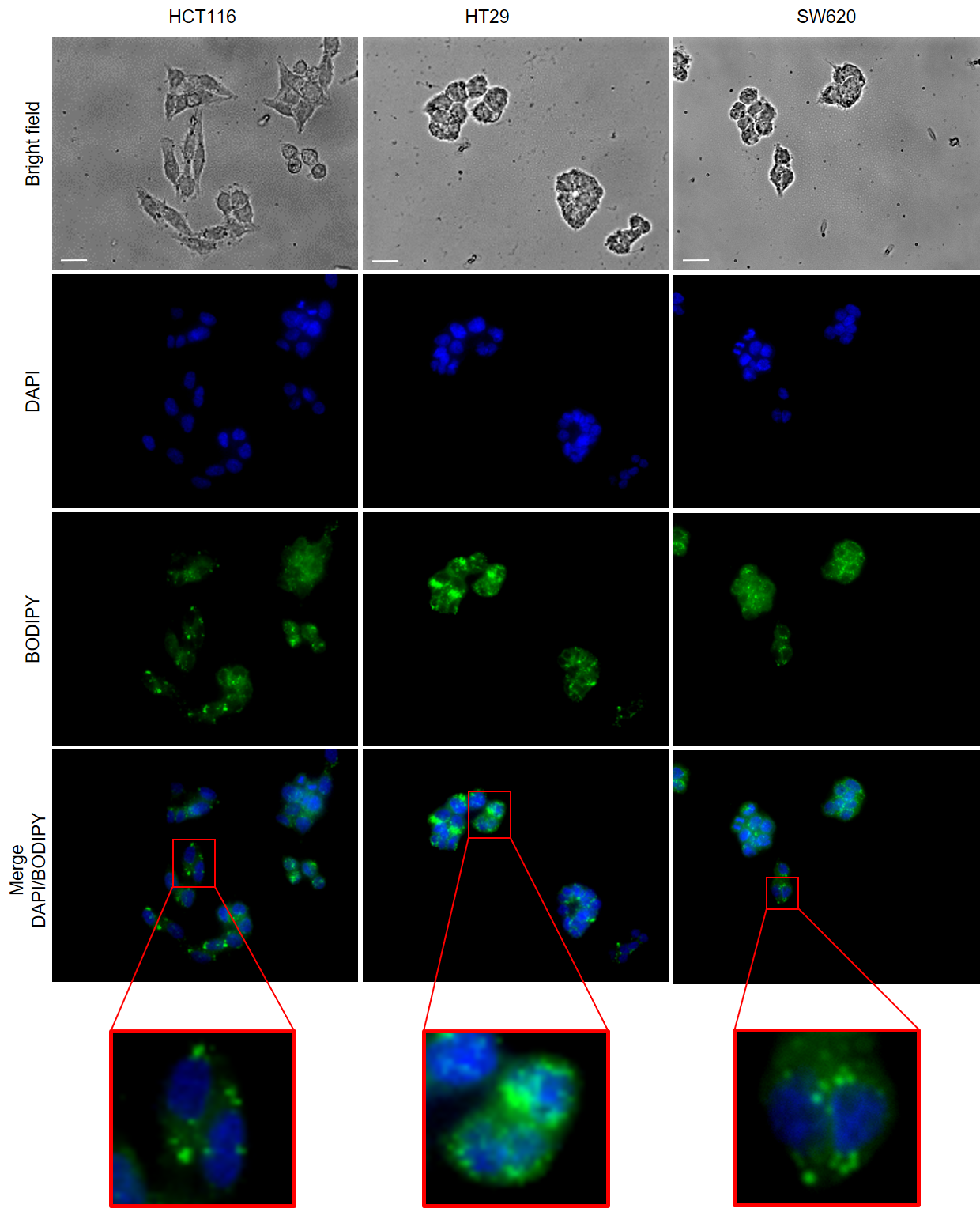


**Supplementary Fig. 5 BODIPY staining of colorectal cancer cell lines, HCT116, HT29 and SW620.** Cells were fixed using PFA, and stained with BODIPY 493/503 (green fluorescence) for lipid and DAPI (blue fluorescence) for nuclei. Scale bar, 10 µm.


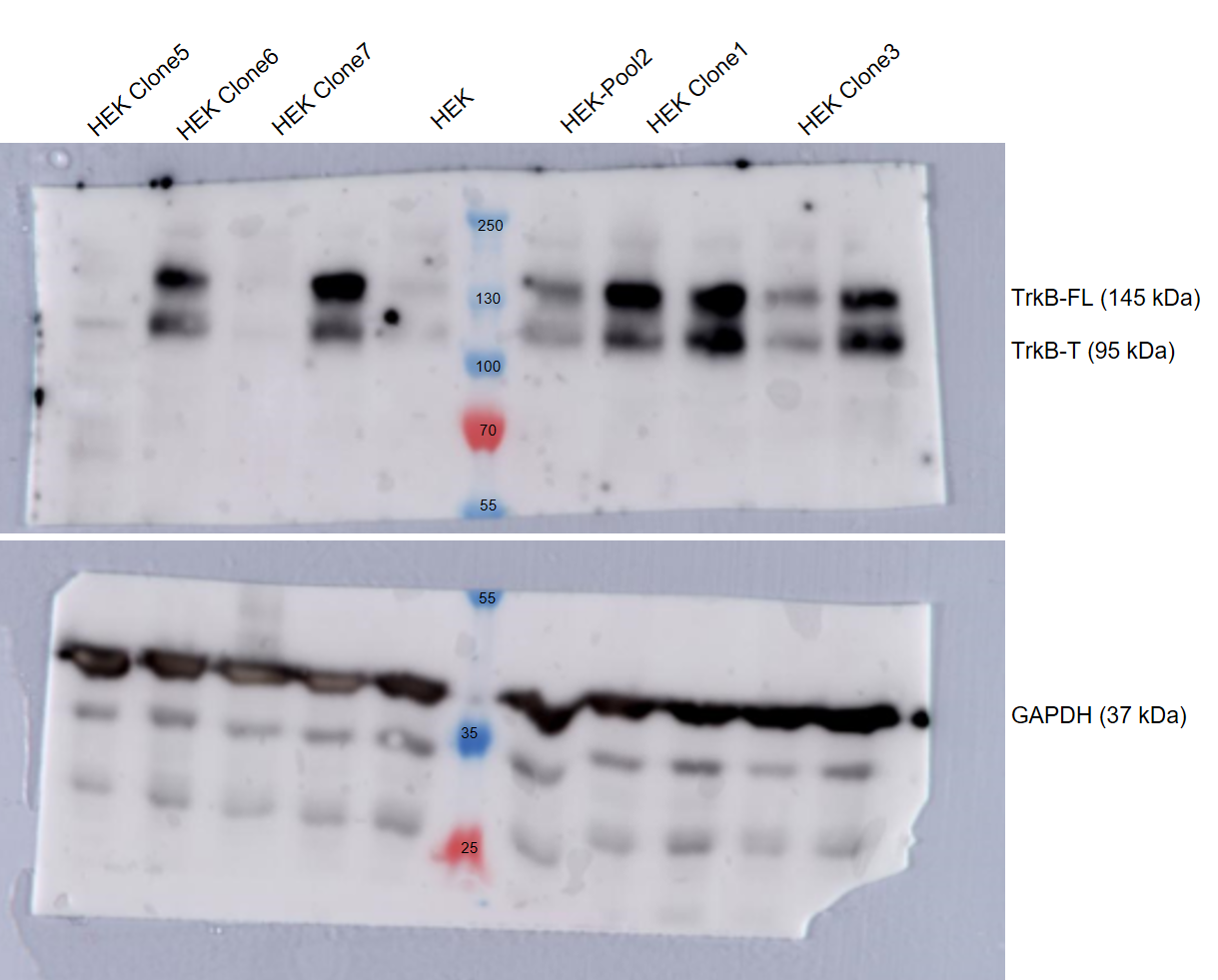


**Supplementary Fig. 6 Full-length of the blots presented in Fig. 3B.** Western-blot analysis of TrkB expression in HEK clones. After protein extraction, western-blot of TrkB was performed with 50 µg of extracted protein.


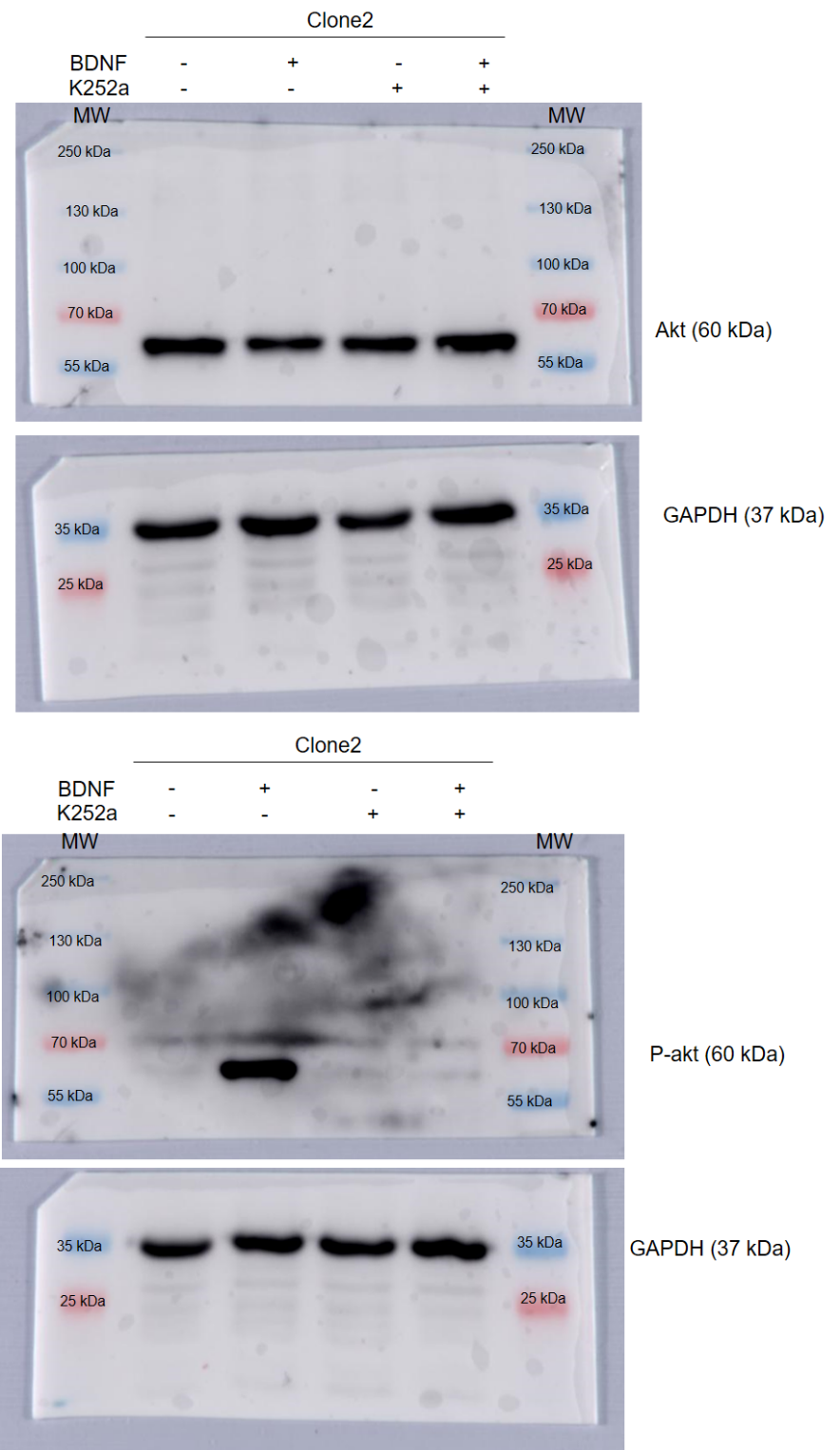


**Supplementary Fig. 7 Full-length of the blots presented in Fig. 3C.** HEK-Clone2 cells were treated with BDNF (75 ng/mL) with or without K252a (100 nM) for 1 hour. Western-blots were realized on 50 µg of extracted protein for A: Akt and P-Akt (phospho-Akt) B: p-TrkB (phosphorylated-TrkB). All western-blots were detected on separate blots with the corresponding specific antibodies.

**
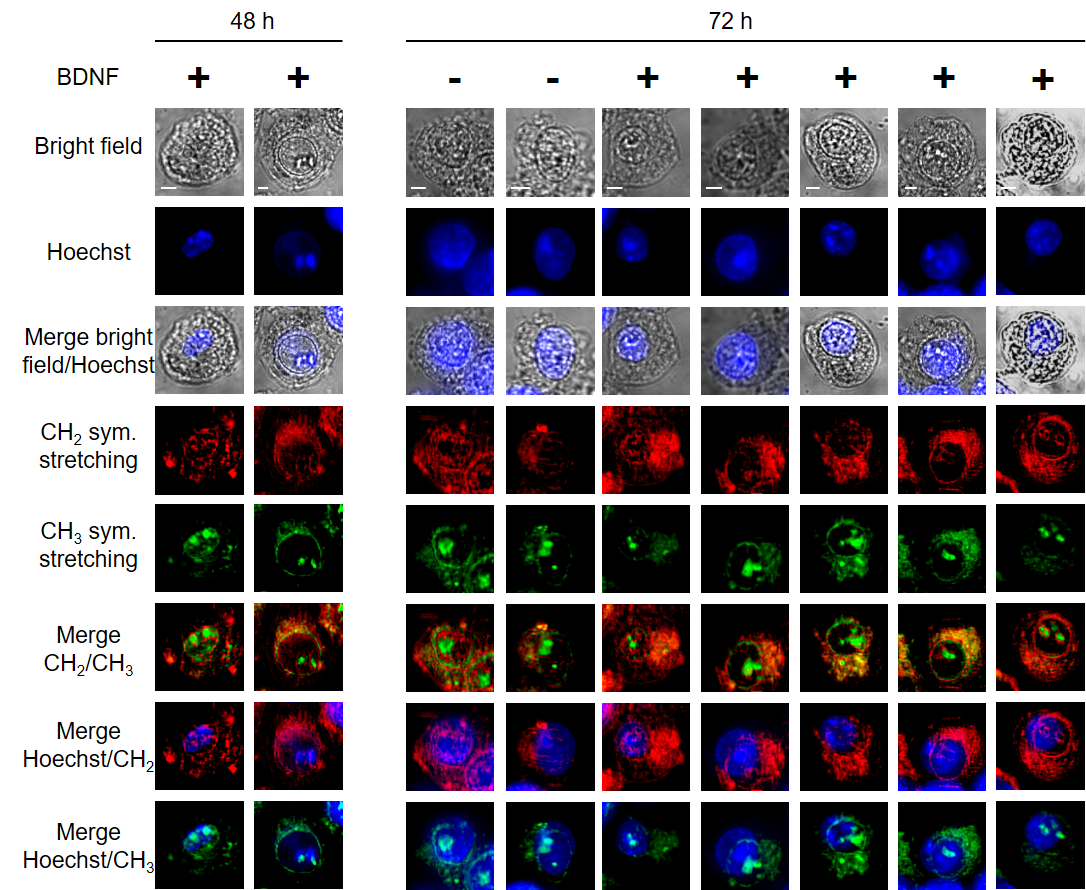
**

**Supplementary Fig. 8 MCARS microspectroscopy of HEK-Clone2 after BDNF-induced TrkB activation.** HEK-Clone2 cells were incubated with, or without BDNF (75 ng/mL), during 48 and 72 hours, the figure includes bright field and fluorescence (Hoechst 33342) images. MCARS images were reconstructed from signal integration at 2850 cm^-1^ (CH_2_ symmetric stretching) and 2930 cm^-1^ (CH_3_ symmetric stretching). Scale bar, 5 µm.


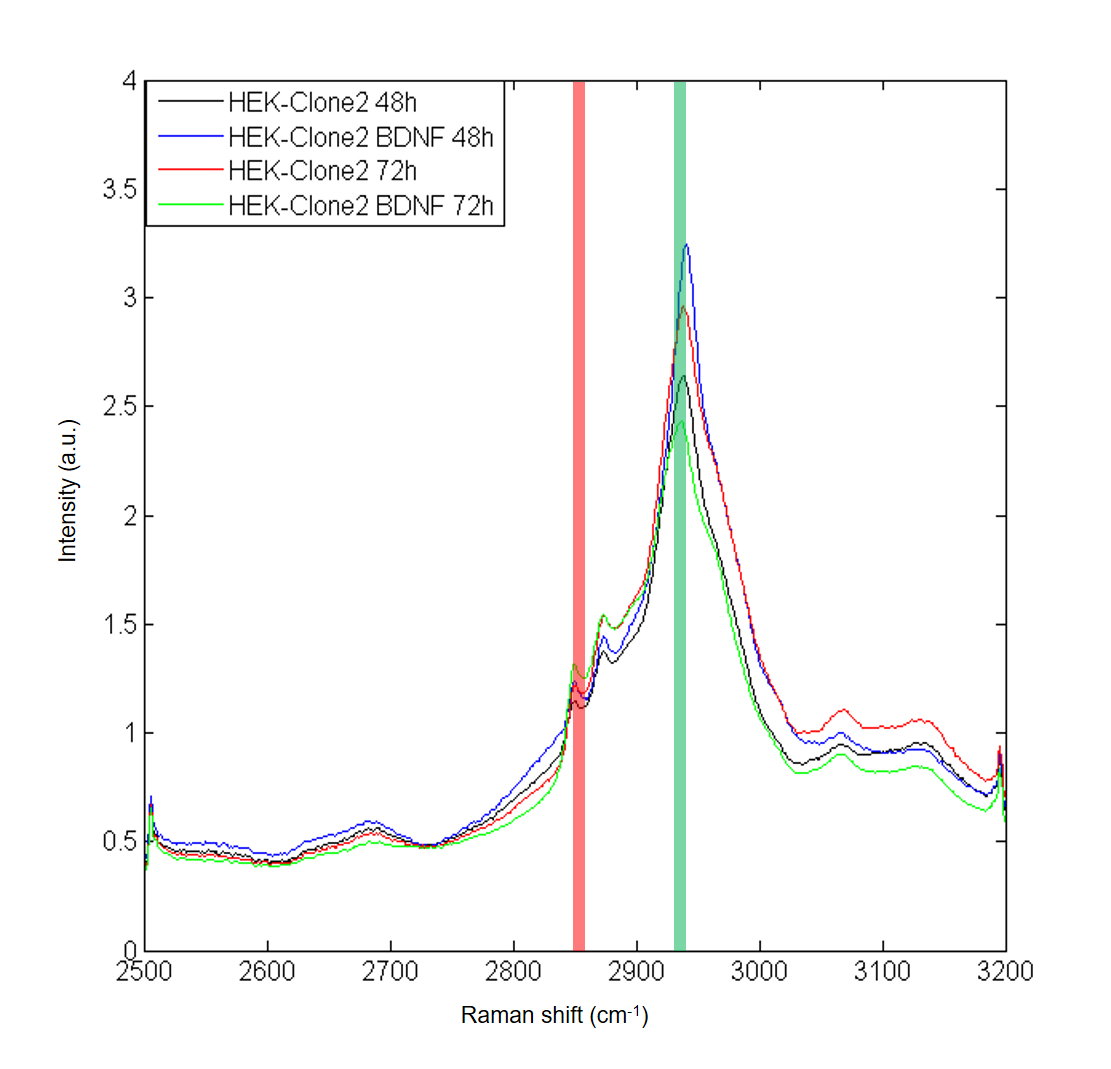


**Supplementary Fig. 9 Mean of standard deviation of the vibrational resonant CARS signal between 2500 and 3200 cm^1^.** computed over the whole region of interest for 3 HEK-Clone2 cells (+/- BDNF) at 48 h, 3 cells HEK-Clone2 cells at 72 h and 6 cells for HEK-Clone2 cell with BDNF at 72 h. CH_2_ and CH_3_ spectral contributions are highlighted in red and green, respectively.


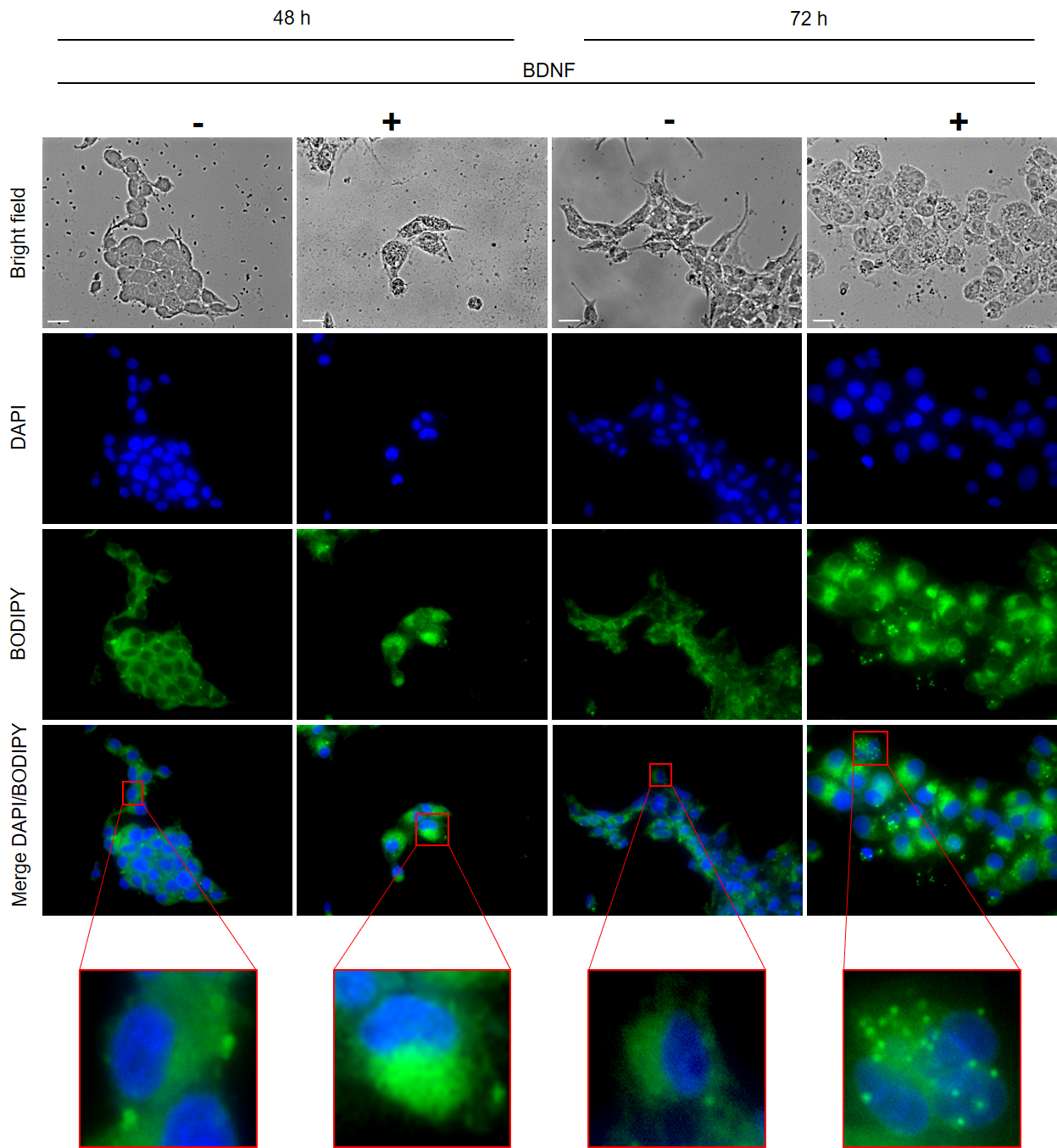


**Supplementary Fig. 10 BODIPY staining of HEK-Clone2 cells after BDNF-induced activation of the TrkB receptor**. HEK-Clone2 cells were treated with, or without BDNF (75 ng/mL), during 48 and 72 hours. Then cells were fixed with PFA, and stained with BODIPY 493/503 (lipid, green fluorescence) and DAPI (DNA, blue fluorescence). Scale bar, 10 µm.


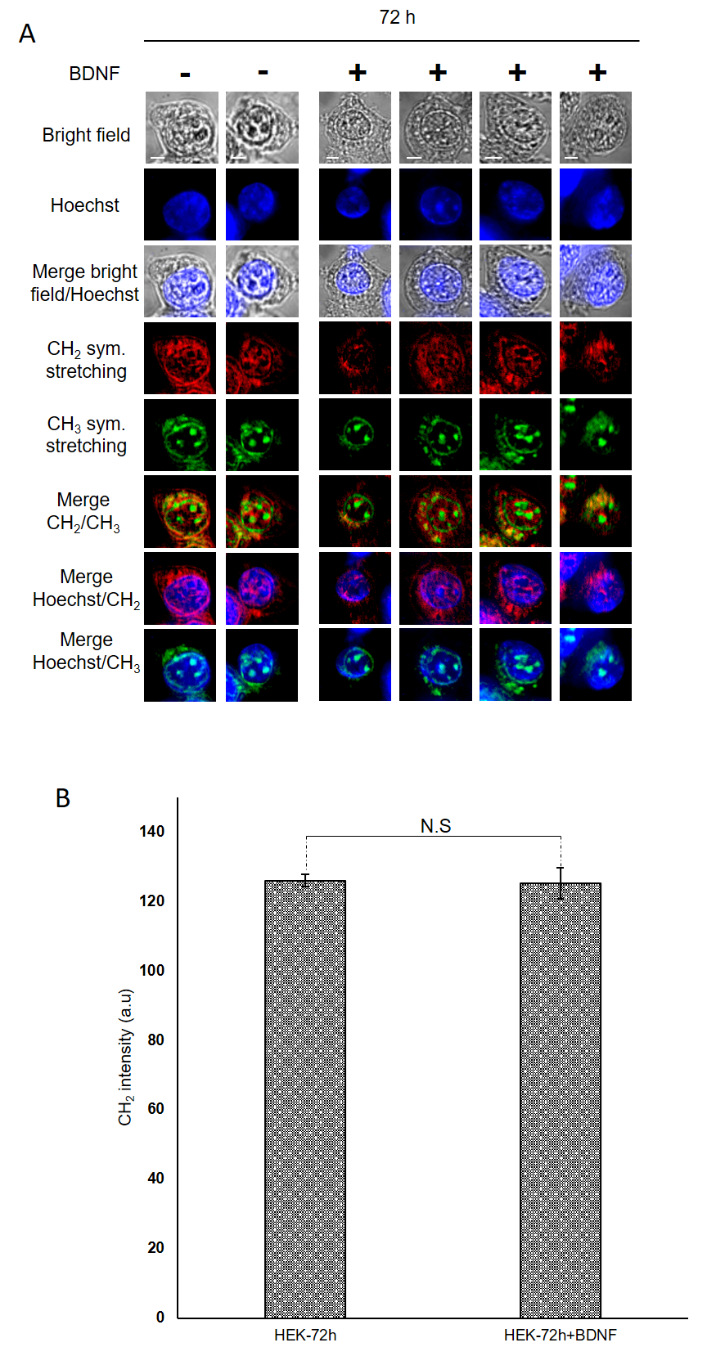


**Supplementary Fig. 11 MCARS microspectroscopy of HEK after BDNF-induced TrkB activation. A.** MCARS analysis of live HEK cells were treated with, or without BDNF (75 ng/mL), during 72 hours, the figure includes bright field and fluorescence (Hoechst 33342) images. MCARS images were reconstructed from signal integration at 2850 cm^-1^ (CH_2_ symmetric stretching) and 2930 cm^-1^ (CH_3_ symmetric stretching). Scale bar, 5 µm. B. Quantification of the CH_2_ signal intensity in HEK cells after treatment with BDNF at 72 hours and MCARS analysis. (N = 3). N.S: Not significant.


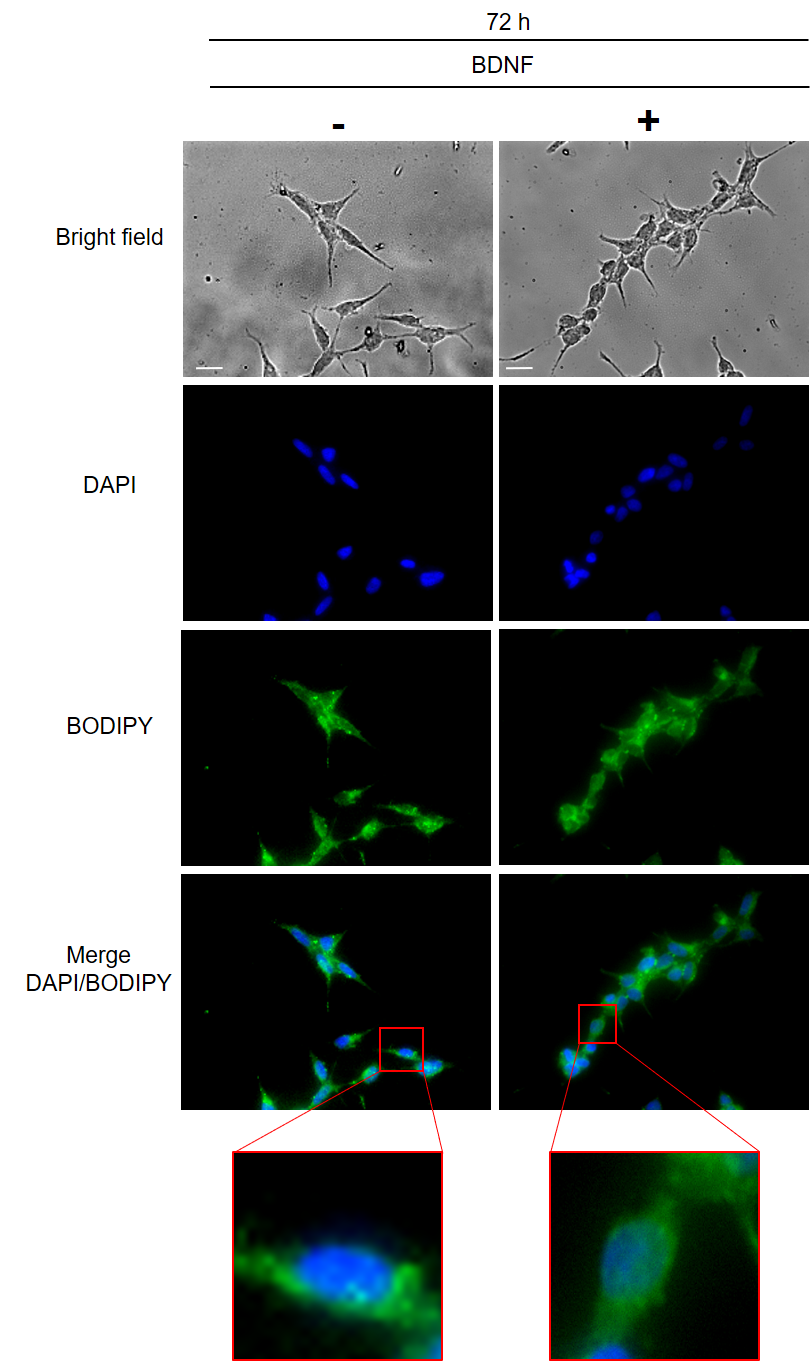


**Supplementary Fig. 12 BODIPY staining of HEK cells after BDNF-induced activation of the TrkB receptor.** HEK cells were treated with, or without BDNF (75 ng/mL), during 72 hours. Then cells were fixed with PFA, and stained with BODIPY 493/503 (lipid, green fluorescence) and DAPI (DNA, blue fluorescence). Scale bar, 10 µm.


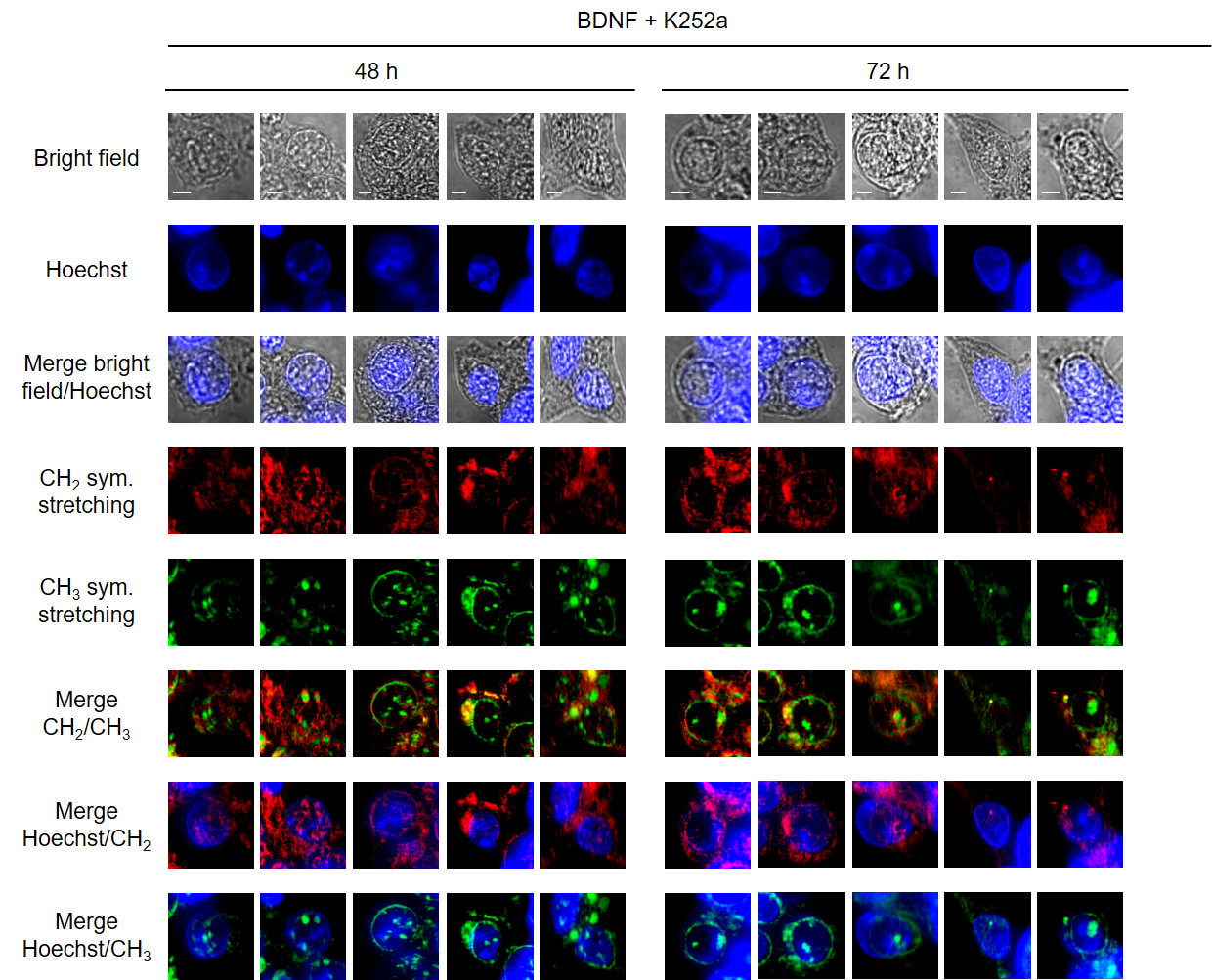


**Supplementary Fig. 13 MCARS microspectroscopy of HEK-Clone2 after K252a inhibition of BDNF-induced TrkB activation.** HEK-Clone2 cells were incubated with BDNF (75 ng/mL) with or without K252a (100 nM), during 48 and 72 hours, the figure includes bright field and fluorescence (Hoechst 33342) images. MCARS images were reconstructed from signal integration at 2850 cm^-1^ (CH_2_ symmetric stretching) and 2930 cm^-1^ (CH_3_ symmetric stretching). Scale bar, 5 µm.


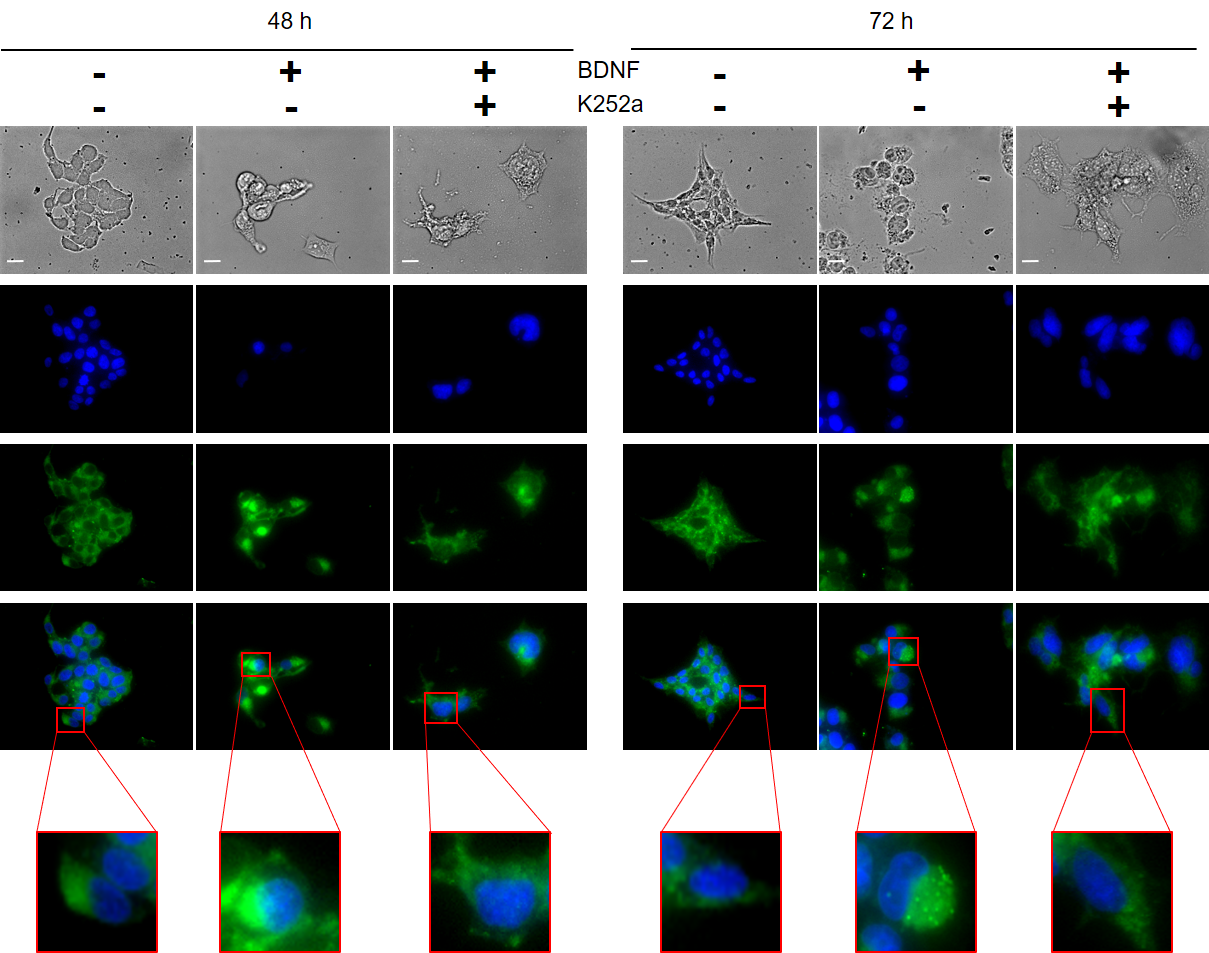


**Supplementary Fig. 14 BODIPY staining of HEK-Clone2 after K252a inhibition of BDNF-induced TrkB activation.** HEK-Clone2 cells were treated with BDNF (75 ng/mL), and/or K252a (100 nM), during 48 and 72 hours. Then the cells were fixed with PFA, and stained with BODIPY 493/503 (lipids, green fluorescence) and DAPI (DNA, blue fluorescence). Scale bar, 10 µm.


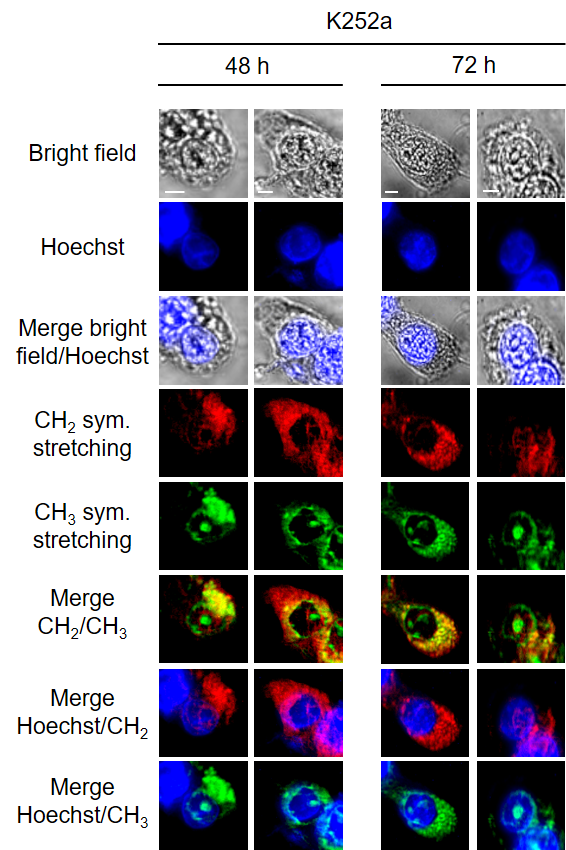


**Supplementary Fig. 15 MCARS microspectroscopy of HT29 cell line after K252a inhibition.** HT29 cell lines were treated with K252a (100 nM), during 48 and 72 hours, the figure includes bright field and fluorescence (Hoechst 33342) images. MCARS images were reconstructed from signal integration at 2850 cm^-1^ (CH_2_ symmetric stretching) and 2930 cm^-1^ (CH_3_ symmetric stretching). Scale bar, 5 µm.


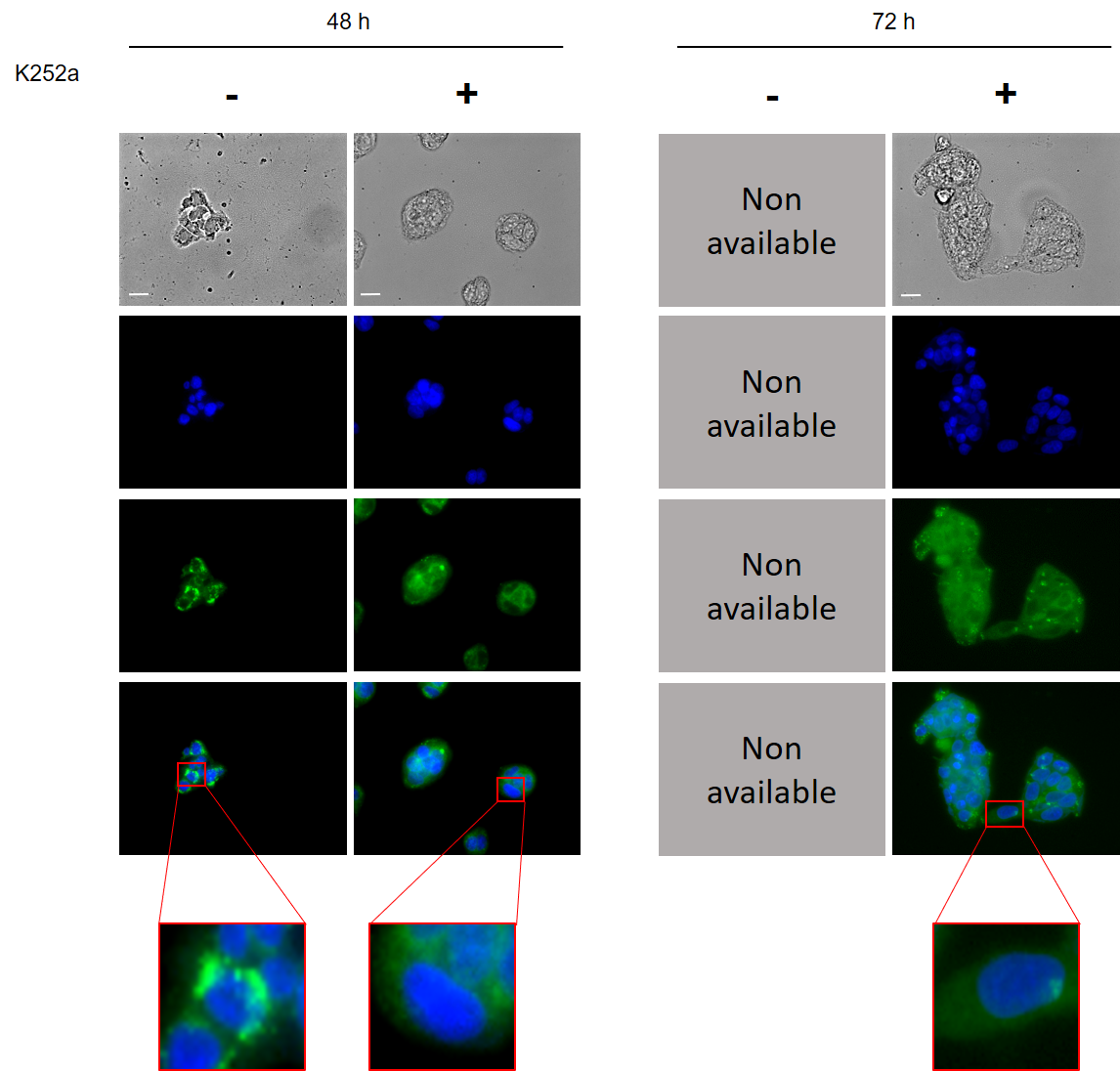


**Supplementary Fig. 16 BODIPY staining of HT29 cell line treated with** K252a inhibition of TrkB expression in HT29 cell line. HT29 cells were treated with or without K252a (100 nM), during 48 and 72 hours. Then cells were fixed using PFA, and stained with BODIPY 493/503 (lipid, green fluorescence) and DAPI (DNA, blue fluorescence). Scale bar, 10 µm.

1. **Supplementary references**

1. Listenberger, L. L., Studer, A. M., Brown, D. A. & Wolins, N. E. Fluorescent Detection of Lipid Droplets and Associated Proteins: Detection of Lipid Droplets by Fluorescent Microscopy. in *Curr. Protoc. Cell Biol.* (eds. Bonifacino, J. S., Dasso, M., Harford, J. B., Lippincott-Schwartz, J. & Yamada, K. M.) 4.31.1-4.31.14 (John Wiley & Sons, Inc., 2016).
